# Recombinant Bovine Herpesvirus Type I Expressing the Bovine Viral Diarrhea Virus E2 Protein Could Effectively Prevent Infection by Two Viruses

**DOI:** 10.3390/v14081618

**Published:** 2022-07-25

**Authors:** Chun-Yu Liu, Hao Guo, Hong-Zhe Zhao, Li-Na Hou, Yong-Jun Wen, Feng-Xue Wang

**Affiliations:** College of Veterinary Medicine, Inner Mongolia Agricultural University, Hohhot 010018, China; chunyuliu_vet@163.com (C.-Y.L.); gh19960627@126.com (H.G.); hongzhezhao@163.com (H.-Z.Z.); hou163@126.com (L.-N.H.)

**Keywords:** BoHV−1, BVDV−1, BoHV−1 gE/E2−Linker−E2^+^, BoHV−1 ΔgE, E2 glycoprotein, signal peptide, neutralizing antibody, vaccine

## Abstract

Bovine respiratory disease complex (BRDC) is a comprehensive disease in cattle caused by various viral and bacterial infections. Among them, bovine herpesvirus type I (BoHV−1) and bovine viral diarrhea virus (BVDV) play important roles and have caused huge financial losses for the cattle industry worldwide. At present, vaccines against BRDC include trivalent attenuated BoHV−1, BVDV−1, and BVDV−2 live vaccines, BoHV−1 live attenuated vaccines, and BoHV−1/BVDV bivalent live attenuated vaccines, which have limitations in terms of their safety and efficacy. To solve these problems, we optimized the codon of the BVDV−1 E2 gene, added the signal peptide sequence of the BoHV−1 gD gene, expressed double BVDV−1 E2 glycoproteins in tandem at the BoHV−1 gE gene site, and constructed a BoHV−1 genetics-engineered vectored vaccine with gE gene deletion, named BoHV−1 gE/E2−Linker−E2^+^ and BoHV−1 ΔgE. This study compared the protective effects in BoHV−1, BoHV−1 ΔgE, BoHV−1 gE/E2−Linker−E2^+^, and BVDV−1 inactivated antigen immunized guinea pigs and calves. The results showed that BoHV−1 gE/E2−Linker−E2^+^ could successfully induce guinea pigs and calves to produce specific neutralizing antibodies against BVDV−1. In addition, after BoHV−1 and BVDV−1 challenges, BoHV−1 gE/E2−Linker−E2^+^ can produce a specific neutralizing antibody response against BoHV−1 and BVDV−1 infections. Calves immunized with this type of virus can be distinguished as either vaccinated animals (gE^-^) or naturally infected animals (gE^+^). In summary, our data suggest that BoHV−1 gE/E2−Linker−E2^+^ and BoHV−1 ΔgE have great potential to prevent BVDV−1 or BoHV−1 infection.

## 1. Introduction

Bovine respiratory disease complex (BRDC) is a comprehensive disease in cattle caused by a variety of viral and bacterial infections, which is causing significant economic losses for the cattle industry in China and worldwide [1,2]. Multiple pathogenic concurrent infections can lead to severe BRDC, among which, the respiratory viral factors involved in BRDC, BoHV−1, and BVDV are important, as these viruses cause immunosuppression. Studies have shown that BVDV infection contributes to BoHV−1 immunosuppression of lymphocytes [3]. Compared with calves infected with BVDV or BoHV−1 alone, after BVDV-infected calves are challenged with BoHV−1, the depletion of CD4+ and CD8+T cells and neutrophils significantly increased, indicating that BVDV can promote BoHV−1 infection and exacerbate the disease process [4,5,6,7,8]. BVDV can induce the apoptosis of B cells and T cells in vivo, activate the apoptosis program after infection with white blood cells, and greatly reduce the number of white blood cells [9]. After BoHV−1 infection in cattle, a lifelong incubation period is established in the Trigeminal ganglia, and, under the pressure of the external environment, the virus is activated and excreted [10]. BVDV infection usually causes acute infection (AI) and persistent infection (PI) in cattle, and persistently infected calves are the main source of BVDV infection because they can shed for life and spread the disease [11,12]. Therefore, both viruses are constantly present in the external environment.

Bovine viral diarrhea virus (BVDV) is the pathogen of bovine viral diarrhea–mucosal disease (BVD–MD), which has caused significant economic losses for the cattle industry worldwide [13,14]. According to the difference between 5′ UTR and autoprotease, BVDV can be divided into two genotypes: type I (BVDV−1) and type II (BVDV−2) [15]. Among the three structural glycoproteins (Erns, E1, and E2) on the outer surface of the virion, E2 is the most abundant surface protein, contains the main BVDV-neutralizing antigenic site, and can induce BVDV-neutralizing antibodies to prevent infection, which is important as it allows for the virus to attach to host cells and can help the virus enter the cell to complete replication and proliferation [16]. E2 forms E2–E2 homo-dimers and E2–E1 hetero-dimers through disulfide bonds, which are present on the surface of the virion [17,18]. During virus assembly, E2 homologous dimers form earlier [19,20]. The expression of the E2 protein and its immunogenicity have been extensively studied in many countries worldwide. Bolin et al. expressed BVDV E2 protein to immunize cattle using the insect cell expression system, which can effectively protect against the challenge of homologous strains [21]. Harpin et al. used the recombinant plasmid containing the E2 gene to immunize cattle, and the results showed limited protection, but the immunized cattle could produce an immune response [22].

BoHV−1 is an enveloped, double-stranded DNA virus belonging to the *Herpesviridae* family, the *Alphaherpesvirinae* subfamily, and the varicella virus genus [23]. The viral genome consists of about 70 encoded proteins, of which 33 structural proteins and more than 15 non-structural proteins have been identified [24]. The gE gene is essential for virus-to-cell transmission and viral anterograde axonal transport [25,26]. In addition, related studies have shown that the virulence of BoHV−1 is reduced after the deletion of the gE gene, the virus in the latent period will not be reactivated, and the transmission of the virus between cells will be affected, which is safe for calves [27,28,29]. Many investigators have deleted the BoHV−1 virulence gene gE, demonstrating that the mutant virus is safe and immunogenic in cattle, and it is a candidate for a locally labeled vaccine [30,31,32,33]. Moreover, gE is a recognized virulence factor for all known members of the *Alphaherpesvirinae* subfamily [32], and has become an ideal target site for BoHV−1 gene deletion [30,34,35].

Conventional vaccines include live attenuated and inactivated vaccines, which could prevent disease but could not differentiate infected from vaccinated animals (DIVA), creating difficulties in the identification, isolation, culling, and eradication of seropositive animals. Gene deletion labeled vaccines have an immunity marking system, which can use deletion genes for the differential diagnosis of wild virus infection and vaccine immunity, which has become the mainstream direction of new vaccine research and development [36,37,38]. Continuous vaccination with live or inactivated gE deficiency vaccines is an effective way to reduce the spread of the virus within herds [39]. At present, in BoHV−1 control programs in some EU countries, vaccines are prepared using highly attenuated BoHV−1 strains. These are called gE-deletion labeled vaccines [40]. Some countries have successfully adopted similar approaches to eradicate pseudorabies (PRV) [41].

In this study, we aimed to use BoHV−1 as a delivery vector for the BVDV−1 subunit vaccine. Initially, we obtained a recombinant BoHV−1 expressing green fluorescent protein (EGFP), named BoHV−1 gE/EGFP^+^. Using reverse screening, a recombinant virus expressing BVDV E2 protein was obtained, named BoHV−1 gE/E2−Linker−E2^+^. The BoHV−1 gE gene deletion virus was named BoHV−1 ΔgE. In this study, E2 mRNA and protein expression were analyzed at the molecular level and protein level, respectively. BoHV−1 gE/E2−Linker−E2^+^ and BoHV−1 ΔgE mutant viruses were immunized to guinea pigs and calves, respectively, and were evaluated in terms of their safety, immunogenicity, and potential serological differentiation. In this study, we provided data on the development of gE deletion (BoHV−1 ΔgE) strains, the BoHV−1 expression of the BVDV−1 E2 protein (BoHV−1 gE/E2−Linker−E2), and the use of inactivated antigens as vaccines. Our results show that BoHV−1 gE/E2−Linker−E2^+^ and BoHV−1 ΔgE viruses are effective vaccine candidates for the prevention of BVDV−1 and BoHV−1 infection when used as inactivated antigens.

## 2. Materials and Methods

### 2.1. Viruses and Cell Lines

BoHV−1 and BVDV were isolated, identified, and stored in the laboratory of Yong-Jun Wen, College of Veterinary Medicine, Inner Mongolia Agricultural University. Bovine Kidney Cell Line (MDBK) was purchased from Kunming Cell Bank (KCB90031YJ). The African Green Monkey Kidney Cell Line (Vero E6) was donated by the Harbin Veterinary Research Institute. MDBK and Vero E6 were cultured in modified Eagle’s medium (Gibco, Grand Island, NE, USA). All media were supplemented with 10% heat-inactivated fetal bovine serum (Gibco, Grand Island, NE, USA, 10099141C) and 1% penicillin and streptomycin (Gibco, Grand Island, NE, USA, 15140122).

### 2.2. Antibody and Adjuvant

BVDV−1 E2 specific monoclonal antibody (mAb) stored in the laboratory. The BVDV FITC conjugate was purchased from VMRD (CJ-F-BVD-10 mL). BVDV−1 mAb E2 gp53 IgG2a Isotype was purchased from VMRD (CATALOG #: 157). BoHV−1 positive serum was stored in the laboratory. Goat Anti-Mouse IgG H&L (FITC) and Goat Anti-Mouse IgG H&L (HRP) were purchased from abcam (ab6785, ab6789). MONTANIDETM ISA 206 VG adjuvant was purchased from Seppic (Shanghai, China). Propolis adjuvant was purchased from Jinhe-Youben (Hohhot, China).

### 2.3. Plasmid Construction

#### 2.3.1. Construction of sgRNA Shear Plasmid

According to the BoHV−1 gene (GenBank: AJ004801.1) of NCBI, upstream and downstream primers targeting gE genes were designed by using Primer 5.0. After sequencing analysis, two pairs of sgRNAs were designed online (http://www.e-crisp.org/E-CRISP/, accessed on 4 July 2020). After denaturing, annealing, and extending, the sgRNA was connected to the pX459 vector (Addgene) to obtain pX459−gE−sgRNA1 and pX459−gE−sgRNA2. Similarly, according to the EGFP gene (GenBank: MN153298.1), two pairs of sgRNAs were designed, denatured, annealed, extended, and connected to the vector pX459 to obtain pX459-EGFP-sgRNA1 and pX459-EGFP-sgRNA2.

#### 2.3.2. Optimization and Synthesis of BVDV−1 E2 Gene

According to the BVDV−1 E2 gene (GenBank: AF083348.1) of NCBI, BVDV−1 E2 protein signal peptide and transmembrane region were analyzed online using signal P 4.1 Server and TMHMM Server, V.2.0. Codon optimization was performed based on mammalian cell preference, with BoHV−1 gD signal peptide gene added at the N terminus and TAA termination codons added at the C terminus. The optimized sequence was sent to Beijing Huada Company.

#### 2.3.3. Construction of the Donor Plasmid

The EGFP was amplified using PCR, and the pCDNA 3.1 vector was cloned by the restriction enzyme sites *Kpn* I and *BamH* I, named pCDNA 3.1−EGFP. The E2 synthesized by Beijing Huada Company was amplified into E2-Linker-E2 using the Over-Lap PCR, and the restriction enzyme sites *Kpn* I and *BamH* I were cloned into the pCDNA3.1 vector, which is pCDNA 3.1-E2-linker-E2. According to the BoHV−1 gE gene, three pairs of primers LgE-F/R, RgE-F/R, and R1gE-F/R were designed. The upstream homologous arm (LgE) was amplified by LgE-F/R. The downstream homologous arms (RgE, R1gE) were amplified by RgE-F/R and R1gE-F/R, respectively. The homologous arms were cloned into pCDNA 3.1−EGFP, pCDNA 3.1, and pCDNA 3.1-E2-linker-E2 vectors to construct the donor plasmid pCDNA 3.1-LgE-EGFP-RgE, pCDNA 3.1-LgE-RgE, and pCDNA 3.1-LgE-E2-linker-E2-R1gE. The plasmids constructed above were identified using enzyme digestion and sequencing. The used primers are shown in Table 1.

### 2.4. Construction of Recombinant Viruses

#### 2.4.1. Screening for BoHV−1 gE/EGFP^+^, BoHV−1 ΔgE and BoHV−1 gE/E2−Linker−E2^+^

Donor plasmids pCDNA3.1-LgE-EGFP-RgE, pCDNA3.1-LgE-RgE, and pCDNA3.1-LgE-E2-linker-E2-R1gE were linearized using *Hind* III or *Xba* I. CRISPR/Cas9 vectors pX459−gE−sgRNA1, pX459−gE−sgRNA2 and linearized fragments were co-transfected into Vero E6 cells at 0.5:0.5:1 using Lipofectamine^®^LTX and Plus Reagent transfected reagent, and the control group was transfected with 2 ug pX459 plasmid. Cell culture fluid was changed after 6 h. After 12 h, BoHV−1 with MOI = 1 was inoculated for 2 h, and the Dulbecco’s modified Eagle’s medium containing 1% low melting point agarose, 1% fetal bovine serum, and 1% penicillin and streptomycin was added. After 72 h, the recombinant virus was screened by inverted fluorescence microscopy and further cloned and multiplied in MDBK cells. In the same way, using CRISPR/Cas9 shear plasmids pX459-EGFP-sgRNA1 and pX459-EGFP-sgRNA2, BoHV−1 ΔgE and BoHV−1 gE/E2−Linker−E2^+^ were obtained by reverse screening based on EGFP.

#### 2.4.2. PCR Identification of Recombinant Viruses

To confirm the successful recombination of BoHV−1 gE/EGFP^+^, BoHV−1 ΔgE, and BoHV−1 gE/E2−Linker−E2^+^, the virus was inoculated with MDBK cells to expand the culture after six generations by plaque cloning. The viral genome was extracted as a template, the BoHV-gE−F/R primers were used for PCR (including the gene sequence of the coding region and the non-coding region), and the PCR conditions included initial denaturation at 94 °C for 2 min, then 94 °C 30 s, 64 °C 30 s, 72 °C 2 min 30 s, for a total of 35 cycles, and finally extended at 72 °C for 10 min. PCR products were sequenced and analyzed. The sequencing positive recombinant viruses were purified by ten generations of plaque cloning to obtain recombinant viruses BoHV−1 gE/EGFP^+^, BoHV−1 ΔgE, and BoHV−1 gE/E2−Linker−E2^+^.

#### 2.4.3. Viral Titer Determination

BoHV−1, BVDV−1 (cp) were inoculated into a confluent monolayer of MDBK cells at MOI = 0.01, and the inoculated cells were freeze–thawed three times at 48 h for BoHV−1 and 72 h for BVDV−1 (cp). The cell supernatants were collected and continuously diluted 10-fold from 10^−1^ to 10^−10^. MDBK cells were inoculated with 0.1 mL/well in 96-well plates. Then, they were placed in an incubator containing 5% CO_2_ at 37 °C. TCID_50_ was calculated using the Reed–Muench method after 4 days.

#### 2.4.4. Comparison of Replication Kinetics

A batch of 12-well plates containing confluent monolayer MDBK cells were inoculated with viruses with MOI = 0.01, and the inoculated cells were frozen thawed three times and collected at 12, 24, 36, 48, 60, and 72 h, respectively. The supernatants were collected using centrifugation and continuously diluted by 10-fold from 10^−1^ to 10^−10^. MDBK cells in 96-well plates were inoculated with serially diluted cell supernatant of 0.1 mL/well. At 4 days after inoculation, TCID_50_ was calculated using the Reed–Muench method. The multi-step growth curve of the virus was plotted by GraphPad Prism 7.0 software (San Diego, CA, USA). All data were shown as averages of three independent experiments.

#### 2.4.5. Virion Structure

BoHV−1, BoHV−1 ΔgE, and BoHV−1 gE/E2−Linker−E2^+^ were purified using sucrose density gradient supercentrifugation or filtered and concentrated using a 3000 MWCO Amicon Ultra-15 centrifugal filter (EMD Millipore, Tullagreen, Ireland). The concentrated viruses were adsorbed on the nickel grids for 5 min, the excess viral liquid on the nickel grids was sucked up with filter paper, the excess staining solution was stained with 2% phosphotungstic acid (pH 7.0) for 30 s, and the excess staining solution was sucked up with filter paper. Virus morphology was observed using JEOL 1230 transmission electron microscopy (JEOL, Tokyo, Japan).

#### 2.4.6. Western Blot

MDBK were inoculated with BoHV−1, BoHV−1 ΔgE, BoHV−1 gE/E2−Linker−E2^+^, and BVDV−1 (cp). Cell samples were collected when the cytopathic effect reached about 90%, and sample concentration was determined with a BCA protein concentration detection kit (Thermo, TK274303, Waltham, MA, USA). Proteins were separated by SDS polyacrylamide gel electrophoresis and transferred to a PVDF membrane. A total of 5% skimmed milk was blocked at room temperature for 2 h. The PVDF membrane was washed three times with TBST, BVDV−1 E2 specific mAb (1:1000 dilution) was incubated at 4 °C for 10 h, and the PVDF membrane was washed three times with TBST. Goat Anti-Mouse IgG H&L (HRP) (1:5000 dilution) was incubated at room temperature for 1 h and the PVDF membrane was washed three times with TBST. The PVDF membrane was observed using ECL chromogenic solution (Thermo, SK255593) in a protein-scanning imager.

#### 2.4.7. RT-PCR

The virus was inoculated into a confluent monolayer of MDBK cells with MOI = 0.01, and the inoculated cells were freeze–thawed three times and collected at 12, 24, 36, 48, 60, and 72 h, respectively. The supernatants were collected by centrifugation, and the DNA was extracted using the viral genome DNA/RNA extraction kit (China, DP315). The primers used in this study were designed according to the optimized sequences of E2 gene (Table 1). Firstly, according to the positive recombinant plasmid concentration and copy number formula, the copy number of the positive standard substance was calculated. Then, 10-times gradient dilutions were performed sequentially, and six consecutive gradients of plasmid DNA with 1 × 10^9^, 1 × 10^8^, 1 × 10^7^, 1 × 10^6^, 1 × 10^5^, and 1 × 10^4^ were selected as templates. The reaction was performed on the applied biosystems QuantStudio 5 (Waltham, MA, USA, A28134) instrument using GoTaq^®^qPCR Master Mix (Madison, WI, USA, A6002) kit, with three replicates for each dilution. Reaction system: SYBR GreenI 10 μL, forward and reverse primers of 0.4 μL for each, DNA template 1 μL, ddH_2_O supplement to 20 μL. Reaction conditions: initial denaturation at 95 °C for 30 s, denaturation at 95 °C for 15 s, annealing and extension at 60 °C for 60 s, a total of 40 cycles, generating standard curves. The copy number of E2 gene in each sample was calculated based on the CT value of the standard curve and plotted by GraphPad Prism 7.0 software (San Diego, CA, USA). All data were shown as averages of three independent experiments.

### 2.5. Vaccine Assessment

#### 2.5.1. Vaccine Preparation

The BVDV−1 (400 mL), BoHV−1 ΔgE (200 mL), and BoHV−1 gE/E2−Linker−E2^+^ (200 mL) antigens were centrifuged at 3000 r/min for 5 min and collected for preparation. The 2-bromoethylamine hydrobromide (BEA, molar mass: 205 g/mol) powder (41 g) was added to the sterilized sodium hydroxide solution of 0.2 mol/L (8 g NaOH added to 1000 mL of injectable water) and fully dissolved so that the BEA concentration was 0.2 mol/L. This was placed in a 37 °C water bath and shaken every 10~15 min to make BEA fully cyclized. After 1 h, cyclization was terminated and Binary Ethylenimine (BEI) was produced at a final concentration of 0.2 mol/L. A total of 8 mL BEI inactivating agents were added to the tested BVDV solution (400 mL), with a final concentration of 0.004 mol/L, and 4 mL BEI inactivating agents were added to 200 mL of the BoHV−1 ΔgE and BoHV−1 gE/E2−Linker−E2^+^, with a final concentration of 0.004 mol/L. After heating to 30 °C, the inactivation started and the temperature was maintained at 30 ± 1 °C. Inactivation lasted for 28 h, and the solution was stirred once every 2~4 h, each time for 10~15 min. Immediately after inactivation, inactivated BVDV−1 was added to an 8 mL filtered solution and sterilized with 50% sodium thiosulfate solution (50 g of injectable water, 50 g sodium thiosulfate), with a final concentration of 1%. BoHV−1 ΔgE and BoHV−1 gE/E2−Linker−E2^+^ were added to 4 mL of filtered sterilized 50% sodium thiosulfate solution (50 g of injectable water, 50 g sodium thiosulfate) to make the final concentration 1%. This was fully and evenly stirred, and stored at 2~8 °C for future use. The antigens and adjuvant 206 were bathed in water at 35 °C for 20 min until the temperature of both antigens and adjuvant reached 35 °C and moved to the biosafety cabinet. The antigens and adjuvant were quickly mixed and poured into the beaker within 5 s. The beaker was covered with a sterilized cloth, magnetic stirring was performed for 15 min, and the emulsion was kept overnight at 2~8 °C to complete the first vaccine preparation. The above operations were repeated for secondary preparation.

#### 2.5.2. Immunoprotection Assay of Guinea Pigs

A total of 45 guinea pigs aged 2~3 months were divided into the immunization group (30 guinea pigs), blank control group (3 guinea pigs for simulated immunization), and challenge control group (12 guinea pigs). The immunization group was divided into five groups and each guinea pig received intramuscular immunization with inactivated antigen emulsified with propolis adjuvant 3 mL. The blank control group and the challenge control group received an intramuscular immunization with DMEM emulsified with propolis adjuvant 3 mL, with booster immunization after 21 days. After 42 days, BoHV−1 and BVDV−1 challenged in the nasal cavity (3 mL) with a viral titer of 10^7^TCID_50_/mL. Guinea pig blood was collected to isolate serum every 7 days for 56 days. The serum was inactivated at 56 °C for 30 min and stored at −20 °C. Clinical evaluation of signs (fever, cough, and temperature) and tissue (blood, nasal swab) collection were performed within 14 days after the challenge.

#### 2.5.3. Immunoprotective Assay of Calves

A total of 24 BoHV−1 and BVDV−1 double-negative calves aged 3~6 months were divided into the immunization group (15), blank control group (3), and challenge control group (six for simulated immunization). The immunization group was divided into five groups and each calf received intramuscular immunization in the neck with 4 mL of inactivated antigen. Immunization was boosted after 21 days. After 42 days, BoHV−1 and BVDV−1 (12 calves challenged with BoHV−1 and the other 9 calves challenged with BVDV−1) were challenged in the nasal cavity (8 mL) with a viral titer of 10^7^ TCID_50_/mL. Calf blood was collected to isolate the serum every 7 days for 56 days. The serum was inactivated at 56 °C for 30 min and stored at −20 °C before use. Body temperature was measured daily for 3 days before BVDV−1 challenge, and its average value was taken as the basal body temperature. Blood was collected daily to measure the number of white blood cells, and the average value was taken as the basal white blood cell number. After challenge, the calves were observed for 14 days, body temperature was measured daily, the number of white blood cells was measured, and nasal swabs were collected daily for pathogen detection. At 3 days before BoHV−1 challenge, body temperature was measured daily, and the average was taken as the basal body temperature. After challenge, the body temperature was measured, and nasal swabs were taken daily for pathogen detection. The immunized calves were euthanized 14 days after challenge, and the control group was euthanized 14 days later due to clinical severity.

#### 2.5.4. Clinical Symptom Score

On immunization day (0 days), day 21, and day 42, the rectal temperature, feed, and water intake of guinea pigs and calves were clinically assessed. After the challenge, clinical symptoms were recorded daily for 14 days. Clinical evaluation included rectal temperature, nasal and eye secretions, difficulty breathing, cough, drowsiness, anorexia, and diarrhea. The clinical score for each animal was calculated based on the criteria listed in Supplemental Table S1.

#### 2.5.5. Histology Score

Percentage of lung peribronchiolar/bronchial infiltrates: 0 = none, 1 = little (less than 25%), 2 = many (25–75%), 3 = all (greater than 75%). Lung bronchiolar/peribronchiolar infiltrates: 0 = none (occasionally mild infiltration), 1 = mild (abnormal, often with intermittent rings), 2 = moderate (full ring or crescent ring), 3 = severe (complete ring). Interstitial pneumonia symptoms: 0 = none, 1 = mild, 2 = moderate, 3 = severe. Small intestine: 0 = intestinal villi and epithelium are intact and histology is normal, 1 = slight submucosa or lamina propria swelling and separation, 2 = moderate submucosa or lamina propria separation, submucosa or basal edema, 3 = severe submucosa or lamina propria edema, local necrosis and exfoliation, 4 = disappearance of intestinal villi with intestinal necrosis.

#### 2.5.6. Hematoxylin and Eosin Staining

After euthanizing guinea pigs and calves, the muscle tissue of immunization sites, lung, and small intestinal tissue were collected and fixed in 10% buffered neutral formalin for 12 h. Depending on the tissue, different concentrations of ethanol (100%, 75%, 50%, and 25%) were used for dehydration treatment for 30 min, and the ethanol and xylene mixture (1:1) liquid was transparently treated. The tissue was treated overnight with paraffin and xylene mixture (1:1) embedded in paraffin. After cutting the tissue into 7 μm sections and staining with hematoxylin and eosin, the pathological and the morphological changes in each tissue were observed under ZEISS microscopy (ZEISS, HAL100, Gottingen, Germany).

#### 2.5.7. Virus Neutralization Assay

Serum was heat-inactivated at 56 °C for 30 min, diluted two times consecutively in DMEM, and added to equal volumes of DMEM diluted BVDV−1 or BoHV−1 containing 200 TCID_50_ respectively, with a final volume of 200 μL of samples, and incubated at 37 °C for 1 h. MDBK were inoculated with a 100 μL neutralizing mixture. Four replicates of each sample were made at 37 °C, with 5% CO_2_ for 2 h, and replaced by DMEM containing 2% FBS for 4 days to observe the cytopathic effect. The 50% neutralizing titer of serum was calculated using the Reed–Muench method.

#### 2.5.8. ELISA

The BVDV−1 Antibody Detection Kit (IDEXX, Q801) and the BoHV−1 gB Antibody Detection Kit (IDEXX, L101) were used to detect virus specific antibodies. All procedures were following the manufacturer’s instructions. BVDV was determined by S/P ratio ≥0.3 as a positive sample and S/P ratio ≤0.5 as a negative sample. BoHV−1 was determined as a positive sample with a blocking rate of ≥55 and a negative sample with a blocking rate of <45.

#### 2.5.9. White Blood Cell Count

To calculate white blood cells in an EDTA whole-blood sample, an automatic hematology analyzer (mindray bc2600, Beijing, China) was used. Blood was collected 3 days (−2, −1, 0 dpc) before the challenge and 1~14 dpc, and the total number of white blood cells was measured and recorded. The average value was taken 3 days (−2, −1, 0 dpc) before challenge as the base number of white blood cells. In addition, a drop in the white blood cell count of more than 15% for a guinea pig and a drop of more than 25% in the white blood cell count for a calf was considered leukopenia [42].

#### 2.5.10. Data Analysis

The experimental data were expressed as mean ± standard deviation. The differences were analyzed using the ANOVA test. *p* <  0.05 was considered significant.

## 3. Results

### 3.1. Screening and Identification of Recombinant Viruses

**Figure 1:**
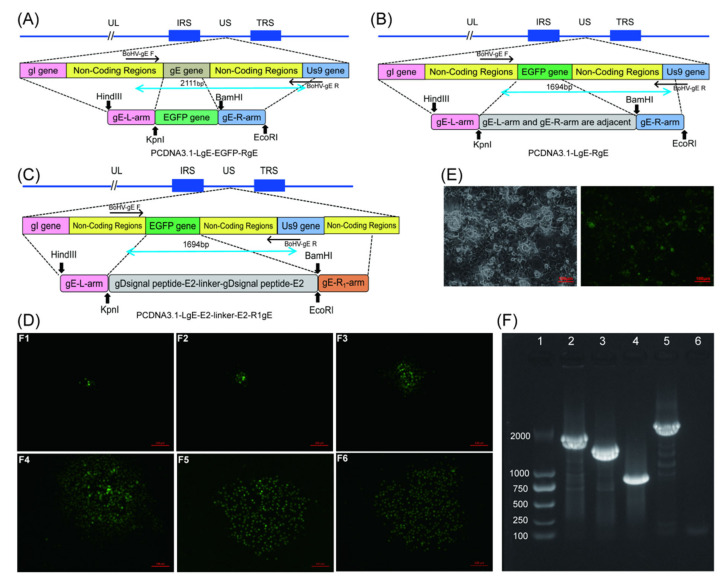
Construction of BoHV−1 donor plasmids and screening of recombinant viruses. (**A**) The sequence information of the pCDNA 3.1-LgE-EGFP-RgE donor plasmid and the recombinant site inserted into the BoHV−1 genome while using CRISPR/Cas9 system to edit BoHV−1; (**B**) pCDNA 3.1-LgE-E2-Linker-E2-R1gE; (**C**) pCDNA3.1-LgE-RgE; (**D**) BoHV−1 gE/EGFP^+^ was successfully obtained. F1 showed the first generation of recombinant virus and screened virus with green fluorescent signals for 6 generations using plaque cloning. (**E**) MDBK were inoculated with BoHV−1 gE/E2−Linker−E2^+^ (MOI = 0.1) and identified by immunofluorescence using BVDV−1 E2 specific mAb 36 h later. The figure on the left shows the same area in the bright field, and the figure on the right shows E2 expression. (**F**) PCR identified BoHV−1. The viral genome was extracted from the expanded cultured viral fluid after six generations by plaque cloning and amplified by PCR using BoHV−1 gE−F/R primers, and the negative control indicated ddH_2_O. Lane 1 is DNA molecular-weight 2000, lane 2 is wtBoHV−1, lane 3 is BoHV−1 gE/EGFP^+^, lane 4 is BoHV−1 ΔgE, lane 5 is BoHV−1 gE/E2−Linker−E2^+^, and lane 6 is negative control.

**Figure 2:**
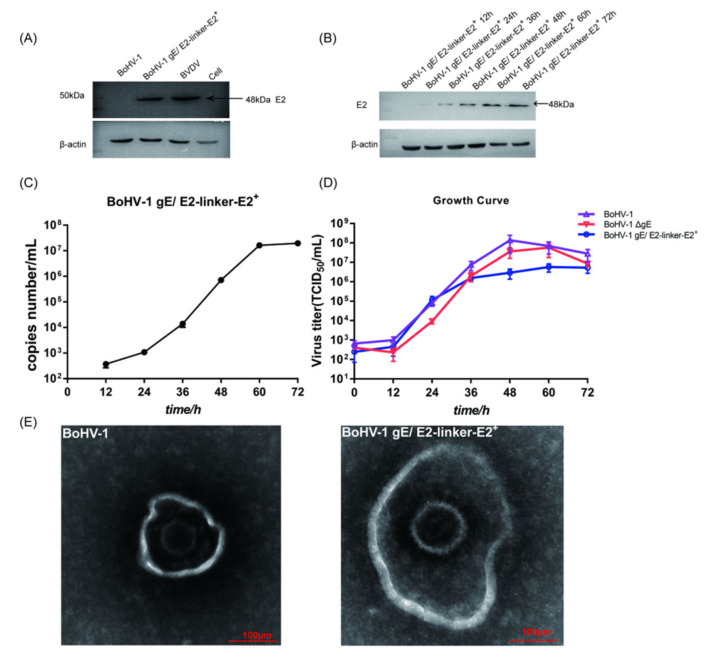
The insertion of the gene did not change the viral replication ability and particle morphology. (**A**) Western blot analysis of BoHV−1 gE/E2−Linker−E2^+^ with chimeric expression of BVDV−1 E2 and E2-Linker-E2 using BVDV−1 E2-specific mAb. (**B**) Evaluation of E2-linker-E2 expression levels of BoHV−1 gE/E2−Linker−E2^+^ after inoculation of MDBK using BVDV−1 E2-specific mAb. Expression of BVDV−1 E2 protein in cells at 12, 24, 36, 48, 60, and 72 h. (**C**) Evaluation of E2-linker-E2 expression at mRNA levels using E2–E2−F/R primers by RT-PCR. Calculation of the copy number of the recombinant virus genome based on the CT value of the standard curve. mRNA expression of the BoHV−1 gE/E2−Linker−E2^+^ genome was shown at 12, 24, 36, 48, 60, and 72 h, and the above experiments were independently replicated three times within each time-period. (**D**) Recombinant viruses were inoculated MDBK (MOI = 0.01). At 12, 24, 36, 48, 60, and 72 h, the virus titer was calculated using the Reed–Muench method, and the virus growth curve was plotted. (**E**) After phosphotungstic acid staining, the morphological characteristics of BoHV−1 and other recombinant viruses were compared using electron microscopy.

Three BoHV−1 gE-specific donor plasmids were successfully designed and constructed: pCDNA 3.1-LgE-EGFP-RgE (Figure 1A) contains the EGFP gene, the LgE (1054 bp) of gE gene, and RgE of the gE gene (748 bp); pCDNA 3.1-LgE-RgE (Figure 1B) contains only LgE (1054 bp) and RgE (748 bp) of the gE gene; and pCDNA 3.1-LgE-E2-Linker-E2-R1gE (Figure 1C) contains the BVDV−1 E2-optimized gene, LgE (1054 bp) of the gE gene, and R1gE (1086 bp) of the gE gene. All plasmid sequences were confirmed to be correct by sequencing. Next, we co-transfected the CRISPR/Cas9 construct pX459−gE−sgRNA1, pX459−gE−sgRNA2, and pCDNA3.1-LgE-EGFP-RgE donor plasmid (linearized with restriction enzyme *Hind* III or *BamH* I) with Vero E6 cells in a ratio of 0.5:0.5:1, and BoHV−1 was inoculated after 12 h. After 72 h, the cytopathic effect and EGFP expression were observed using inverted fluorescence microscopy. The presence of the cytopathic effect and green fluorescence signal in cells was considered to show EGFP positive recombinant viruses, and the suspected virus was harvested and generated on MDBK cells, which were observed again 48 h later using an inverted fluorescence microscope. EGFP-positive recombinant virus was performed for six generations by plaque cloning (Figure 2D). In the same way, cells with a cytopathic effect and no green fluorescence signal were considered to be present in EGFP-negative recombinant viruses, and we screened recombinant viruses BoHV−1 gE/EGFP^-^ and BoHV−1 gE/E2−Linker−E2^+^ using donor plasmid pCDNA3.1-LgE-RgE and pCDNA3.1-LgE-E2-Linker-E2-R1gE. The suspected positive result was performed for six generations using plaque cloning. At the same time, the recombinant virus was identified by PCR using BoHV−1 gE−F/R primers. The expected product of the parental virus BoHV−1 was 2111 bp (Figure 1F, lane 2), the expected product of the recombinant virus BoHV−1 gE/EGFP^+^ was 1694 bp (Figure 1F, lane 3), the expected product of the recombinant virus BoHV−1 ΔgE was 981 bp (Figure 1F, lane 4), and the expected product of the recombinant virus BoHV−1 gE/E2−Linker−E2^+^ was 2624 bp (Figure 1F, lane 5). The above PCR products were sequenced, the integrity of the chimeric gene sequence and its insertion at the gE deletion site were verified, and the gE gene was successfully replaced by the complete EGFP and E2-Linker-E2 genes. In addition, to verify the expression of the E2 gene in the inoculated cells, the recombinant virus BoHV−1-gE/E2-Linker-E2^+^ (MOI = 0.1) was inoculated using MDBK cells. These were verified using a BVDV−1 E2 specific mAb, and we found that BoHV−1 successfully expressed the BVDV−1 E2 gene as a vector (Figure 1E).

### 3.2. In Vitro Characterization of BoHV−1 gE/E2−Linker−E2^+^ and BoHV−1 ΔgE

In this study, the expression of E2-linker-E2 was verified by SDS-PAGE/western blot after cells were inoculated with BoHV−1 gE/E2−Linker−E2^+^. To this end, the E2 expressions of BVDV−1 and BoHV−1 gE/E2−Linker−E2^+^ were verified. BVDV−1 E2 specific mAb identified protein bands of approximately 48 kD, consistent with the size of BVDV−1 (Figure 2A). As expected, BVDV−1 E2 specific mAb did not bind to the cell lysate that was inoculated with BoHV−1 or any antigens, and the E2-linker-E2 expressed in this study have same size compared to the BVDV−1 E2 band. The gray value of BoHV−1 gE/E2−Linker−E2^+^ and BVDV virus E2 expression was analyzed by Image J software, the gray value of BVDV−1 E2 protein was 64199, and the gray value of BoHV−1 gE/E2−Linker−E2^+^ E2 protein was 42489. The expression of recombinant virus E2 protein could reach 66% of the E2 protein expression of BVDV−1 at the same loading concentration. MDBK were inoculated with BoHV−1 gE/E2−Linker−E2^+^ (MOI = 0.01) and cell lysates were collected at 12, 24, 36, 48, 60, and 72 h after inoculation, using the above method to verify the expression of E2-linker-E2. The results showed that the expression of E2-linker-E2 could be vaguely detected 24 h after inoculation, and E2-linker-E2 expression accordingly increased as the time of infection increased and peaked at 60 h (Figure 2B). To verify whether the protein expression level of E2-linker-E2 was consistent with the mRNA level, we used E2–E2−F/R fluorescence quantitative primers to analyze mRNA changes in the E2 protein in BoHV−1 gE/E2−Linker−E2^+^ (MOI = 0.01) after inoculating MDBK at 12, 24, 36, 48, 60, and 72 h. The results showed that the number of E2-linker-E2 copies also increased with time, reaching a peak at 60 h, consistent with protein levels (Figure 2C). To confirm whether BoHV−1 mutations affect their replication properties, the proliferation of recombinant and parental virus was assessed in MDBK. The results showed that the replication rate of the BoHV−1 ΔgE and BoHV−1 gE/E2−Linker−E2^+^ was not significantly different from that of BoHV−1 within 36 h (Figure 2D). The replication rate of the BoHV−1 gE/E2−Linker−E2^+^ was significantly different from that of BoHV−1 at 48 h (Figure 2D). All viruses reached the titer peak at 60 h (Figure 2D). The growth trend of the two recombinant viruses was similar to that of the parent virus. However, the deletion of the BoHV−1 gE gene and the tandem expression of E2-linker-E2 did not affect the effective replication ability of the virus in cells in vitro, so the virus could be used as a candidate vaccine strain. To detect whether the insertion of an E2-linker-E2 changed the virus morphology, the morphological characteristics of the BoHV−1 and the BoHV−1 gE/E2−Linker−E2^+^ were observed using electron microscopy. Mean ± standard deviation analysis showed that the average diameter of BoHV−1 and BoHV−1 gE/E2−Linker−E2^+^ particles was 201 ± 2 nm and 210 ± 3.27 nm. The average diameter of the mutant virus was not significantly different from that of the parental virus.

### 3.3. Immunoprotection Assay of Guinea Pigs

#### 3.3.1. BoHV−1 gE/E2−Linker−E2^+^ Immunization Group Could Produce Specific Serum Neutralizing Antibodies against BVDV−1

**Figure 3:**
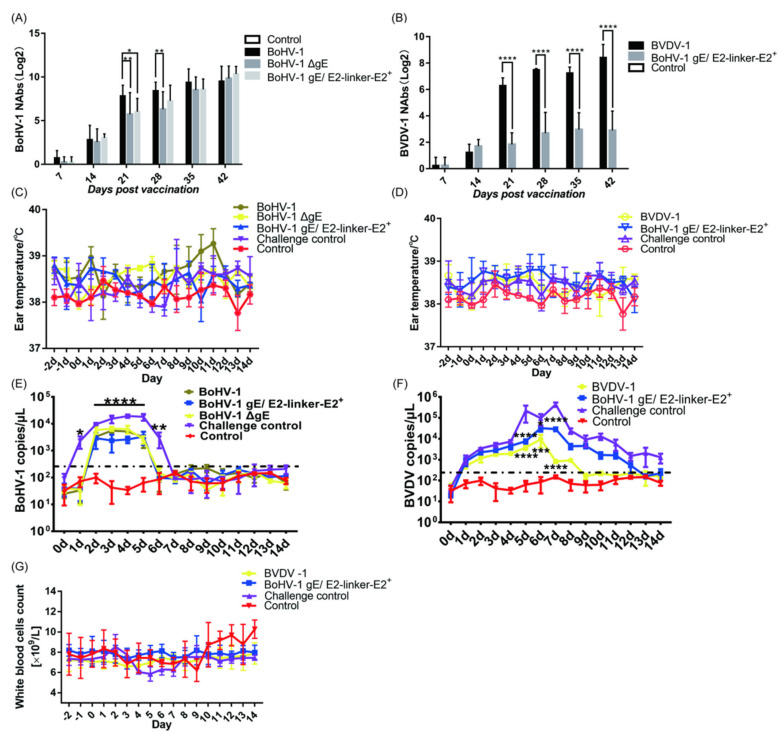
Immunogenicity and protective experiments of BoHV−1 gE/E2−Linker−E2^+^ and BoHV−1 ΔgE on guinea pigs. Immunogenicity experiment: intramuscular immunization of inactivated antigen, second immunization 21 days after the first immunization. Serum samples were collected to detect specific neutralizing antibodies 7 days after the initial immunization until day 42. (**A**) BoHV−1 specific neutralizing antibody. (**B**) BVDV−1 specific neutralizing antibody. Protective experiment: after 42 d immunization, guinea pigs were challenged with BoHV−1 or BVDV−1. (**C**) Changes in the body temperature of each group of guinea pigs after the BoHV−1 challenge. (**D**) Changes in the body temperature of guinea pigs in each group after the BVDV−1 challenge. (**E**) Viral load in guinea pigs’ nasal swabs after BoHV−1 challenge and the average copy number in 1 μg of total DNA were analyzed. The dotted line separates the negative result from the positive result (the minimum copy number detected is 3.75 × 10^2^ copies/μL). (**F**) Viral load in guinea pig anal swab after the BVDV−1 challenge; the average copy number in 1 μg total RNA was analyzed. The dotted line separates the negative result from the positive result (the minimum copy number detected is 2.25 × 10^2^ copies/μL). (**G**) After immunization of guinea pigs, the average number of white blood cells after BVDV−1 challenge in each group of guinea pigs. The above experimental data are expressed in mean ± SD. Univariate (ANOVA) was used to analyze whether the difference was significant. *p* < 0.05 is considered significant. Mark * is *p* < 0.05, mark ** is *p* < 0.01, mark *** is *p* < 0.001, mark **** is *p* < 0.0001.

To determine whether there is a difference in immunogenicity between the recombinant viruses and their parent virus on guinea pigs and whether antibodies produced by BoHV−1 gE/E2−Linker−E2^+^ present the ability to neutralize BVDV−1, we mixed inactivated antigens with propolis adjuvants to immune guinea pigs. On day 7 of immunization, the highest serum neutralizing titer of three guinea pigs in the BoHV−1 immunization group reached 1:2 (Figure 3A). In BoHV−1 gE/E2−Linker−E2^+^ and BoHV−1 ΔgE immunization groups, only one guinea pig’s serum neutralizing titer could reach 1:2. As expected, the guinea pigs in the challenge control group did not produce any neutralizing antibodies (Figure 3A). After 21 days, the average serum neutralizing titer of guinea pigs in the BoHV−1 immunization group reached 309.81. The average serum neutrality titer of the BoHV−1 ΔgE immunization group reached 161.02, which was significantly different from that in the BoHV−1 immunization group. The average neutralizing titer of the BoHV−1 gE/E2−Linker−E2^+^ immunization group reached 94.64, which was significantly different from that of the BoHV−1 immunization group. On day 28, the average serum neutralizing titer of guinea pigs in the BoHV−1 immunization group reached 413.55, which was three times that of the BoHV−1 ΔgE immunization group. On day 42, the average serum neutralizing titer of guinea pigs in the BoHV−1, BoHV−1 ΔgE, and BoHV−1 gE/E2−Linker−E2^+^ immunization groups reached 1188.20, 1264.16, and 1501.19, respectively (Figure 3A). The final neutralizing titer of the BoHV−1 immunization group was not significantly different from that of the BoHV−1 gE/E2−Linker−E2^+^ and BoHV−1 ΔgE immunization groups. Importantly, all viruses can cause intense neutralizing activity in guinea pigs, producing a final titer comparable to the neutralizing titer induced by BoHV−1. Deletion of the BoHV−1 gene gE and the insertion and expression of exogenous proteins did not affect its immunogenicity. The serum neutralizing titer of guinea pigs in the BVDV−1 and BoHV−1 gE/E2−Linker−E2^+^ immunization groups also reached 1:2 on 7 days after immunization (Figure 3B). The average serum-neutralizing titers of guinea pigs after 42 days were highest, at 413.55 and 11.75, respectively. The neutralizing titer of the BoHV−1 gE/E2−Linker−E2^+^ immunization group was significantly different from that of the BVDV−1 immunization group (Figure 3B). The E2-Linker-E2 protein expressed by BoHV−1 gE/E2−Linker−E2^+^ could make guinea pigs produce serum neutralizing antibodies against BVDV−1 infection.

#### 3.3.2. After BoHV−1 and BVDV−1 Challenge, the Time and Level of Viral Shedding of Immunized Guinea Pigs Were Less than Those of the Challenge Control Group

At 3 weeks after the second immunization, BoHV−1, BoHV−1 ΔgE, BoHV−1 gE/E2−Linker−E2^+^, and challenge control groups were intranasally challenged with BoHV−1. The body temperature of all immunization groups was within the normal range, with no significant difference (Figure 3C). One guinea pig in the BoHV−1 challenge control group had white secretions in the nasal cavity, which lasted for about 3 days. Three guinea pigs presented with dark yellow secretions in the nasal cavity and eyes for about 6 days. After a challenge with BoHV−1 in BoHV−1, BoHV−1 ΔgE, and BoHV−1 gE/E2−Linker−E2^+^ immunization groups, only one guinea pig in the BoHV−1 immunization group had white secretions in the nasal cavity, which lasted for about 2 days. Guinea pigs in the above immunization group were given nasal swabs for antigen detection using BoHV−1 gE−F/R primers and only one guinea pig in the challenge group was positive for PCR on day 4; the rest of the PCR tests were negative (Supplementary Table S2). We also used the fluorescence quantitative primer gB−F/R to detect the viral shedding of BoHV−1 after the challenge. BoHV−1 could not be detected in the challenge control group on day 7 and BoHV−1 could not be detected in the immunization group on day 6 (Figure 3E). Comparing all immunization groups with the challenge control group, there was a significant difference on day 1 after the challenge. The difference was significant from 2 to 5 days, and the difference was significant on day 6. The virus copy number for guinea pigs immunized with BoHV−1 gE/E2−Linker−E2^+^, BoHV−1, and BoHV−1 ΔgE inactivated antigens was significantly different from that of the challenge control group (Figure 3E). The results showed that the time and level of viral shedding of guinea pigs challenged with BoHV−1 gE/E2−Linker−E2^+^, BoHV−1, and BoHV−1 ΔgE decreased compared to those of the challenge control group when challenged with external BoHV−1.

At 3 weeks after the second immunization, BoHV−1 gE/E2−Linker−E2^+^, BVDV−1, and the challenge control group were challenged in the nasal cavity with BVDV−1. The body temperature of all immunization groups was within the normal range, and there was no significant difference (Figure 3D). Three guinea pigs in the challenge control group had white secretions in the nasal cavity, which lasted for about 8 days. Two guinea pigs presented with dark yellow secretions in the nasal cavity and eyes for about 6 days. Guinea pigs in the above immunization groups had anal swabs taken for antigen detection using 5′ UTR−F/R primers, and all guinea pigs in the challenge group were positive for PCR on days 1~14. Guinea pigs in the BVDV−1 immunization group were positive for PCR on day 1~6, and those in the BoHV−1 gE/E2−Linker−E2^+^ immunization group were positive for PCR on day 1~10. The rest of the PCR tests were negative (Supplementary Table S3). We also used the fluorescence quantitative primer BVDV−F/R to detect viral shedding after the BVDV−1 challenge. Viral shedding could be detected in all the control groups from days 1 to 14, while BVDV−1 could not be detected in the BVDV−1 immunization group on day 8, and BVDV−1 could not be detected in the BoHV−1 gE/E2−Linker−E2^+^ immunization group on day 13 (Figure 3F). Comparing all immunization groups with the challenge control group, the difference was significant on days 5 and 7, while the difference in the BoHV−1 gE/E2−Linker−E2^+^ immunization group was significant on day 6, and the difference in the BVDV−1 immunization group was significant on day 6. The virus copy number of guinea pigs immunized with BoHV−1 gE/E2−Linker−E2^+^ and BVDV−1 inactivated antigens was significantly different from that of the challenge control group.

#### 3.3.3. After the BVDV−1 Challenge, Mild and Transient Leukopenia Was Observed in the Guinea Pigs in the Challenge Group, While No Leukopenia Occurred in the Immunization Group

The total number of white blood cells was measured and recorded 3 days before the challenge (−2, −1, 0 dpc) and 14 days after the challenge. According to the criteria for a percentage decrease in white blood cell count described in the Materials and Methods section, the results showed that two guinea pigs in the challenge control group had symptoms of leukopenia from days 4~6 (Figure 3G). The percentage decrease in white blood cell count for guinea pigs in the challenge control group was 16.96%, 14.29%, 14.29%, and 12.95% from days 4~7, respectively. The other three guinea pigs in the challenge control group all had leukopenia from days 4~7 (Supplementary Table S4). It is worth noting that the maximum percentage decrease in guinea pigs in the challenge control group was between 15.67 and 38.36%, and the average percentage decrease in the challenge control group was 17.94% (Figure 3G). According to the percentage decrease in the white blood cell count, none of the six guinea pigs in the BVDV immunization group had leukopenia symptoms (Supplementary Table S4). One of the guinea pigs in BoHV−1 gE/E2−Linker−E2^+^ immunization group showed symptoms of leukopenia, which decreased by 25.29%, 15.95% and, 17.12% respectively, from the 3rd to 5th days. On day 6, the number of white blood cells rose by 0.038%. Another guinea pig only had leukopenia symptoms on day 7 (17.20%), day 8 (7.53%), and day 9 (11.83%); the reduction was within 15%. The number of white blood cells on day 10 increased by 3.23%, showing no difference from the blank control group, and the number of white blood cells decreased from 8.566 × 10^9^ to 7.1 × 10^9^ on day 7, remaining within the normal range. After the BVDV−1 challenge, mild and transient leukopenia occurred in guinea pigs in the challenge control group, while no leukopenia occurred in all immunization groups.

#### 3.3.4. According to the Histopathological Lesions of the Guinea Pig Lungs, the Immunization of Inactivated Antigen Groups Showed a Good Protective Effect, and the Lung Lesions Were Reduced Compared with the Challenge Control Group

**Figure 4:**
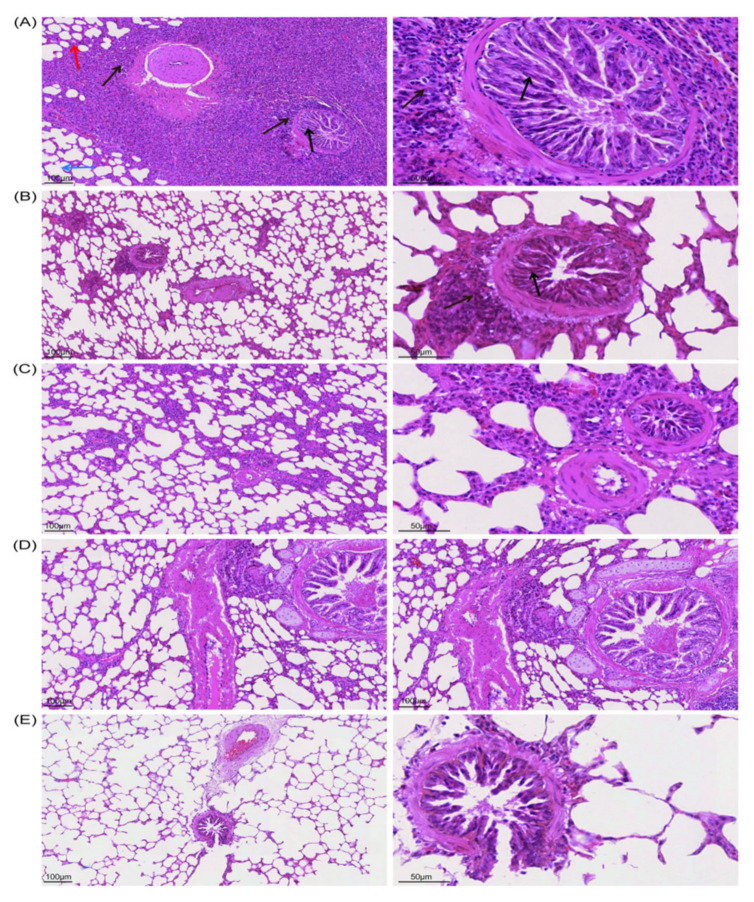
Histopathology of guinea pig lung slices. (**A**) Challenge control group (10 × 20), black arrows show bronchi were compressed, with bulging, bronchospasm, contraction; brown arrows show peripheral lymphocytes and monocyte infiltration, peribronchitis or tubular formation; red arrows show narrowing of alveolar septum and emphysema. (**B**) BoHV−1 immune group (10 × 20). (**C**) BoHV−1 ΔgE immunization group (10 × 20). (**D**) BoHV−1 gE/E2−Linker−E2^+^ immunization group (10 × 20). (**E**) Blank control group (10 × 20).

After 14 days of BoHV−1 challenge, guinea pigs were euthanized, lung slices were taken, and HE staining results showed that the most consistent lesion in all guinea pig lungs was increasing scattered or diffuse peribronchial lymphocytosis, especially in the challenge control group, and there were varying numbers of monocytes and lymphocytes around the bronchi and alveoli. In the challenge control group (Figure 4A), the lung septum of guinea pigs was extensively thickened, the alveolar septum widened, the alveoli became smaller and parenchyma, the bronchi was compressed, there was bulging, bronchospasm, contraction, and varying numbers of lymphocytes and monocytes were infiltrating and proliferating, forming in peribronchitis or tubular manner, which is a typical symptom of interstitial pneumonia. This was accompanied by severe lesions and narrowing of the lung septum, as well as emphysema. In the BoHV−1 (Figure 4B) and BoHV−1 gE/E2−Linker−E2^+^ (Figure 4D) immunization groups, the lesions were mild, with a slight infiltration of inflammatory cells around the bronchi, and clear alveolar space and intact overall lung structure, which was not significantly different from the blank control group (Supplementary Table S9). The BoHV−1 ΔgE immunization group (Figure 4C) showed slightly more severe pathological changes than the BoHV−1 and BoHV−1 gE/E2−Linker−E2^+^ immunization groups. In the BoHV−1 ΔgE immunization group, the alveolar and peribronchronchial lymphocytes were infiltrated and scattered. In summary, the lung lesions of guinea pigs in all immunization groups were significantly different from those of guinea pigs in the challenge control group (Supplementary Table S9), indicating that the deletion of virulence genes and the expression of exogenous proteins did not affect the immunogenicity of the virus and could protect guinea pigs from BoHV−1 challenges.

### 3.4. Immunogenicity and Protective Experiments of BoHV−1 gE/E2−Linker−E2^+^ and BoHV−1 ΔgE on Calves

#### 3.4.1. The Average Titer of Neutralizing Antibody in Serum of BoHV−1 in the BoHV−1 Group Was Not Significantly Different from That of BoHV−1 ΔgE and BoHV−1 gE/E2−Linker−E2^+^ Groups 42 Days after Immunization of Inactivated Antigen, and the BoHV−1 gE/E2−Linker−E2^+^ Group Could Produce Specific Serum-Neutralizing Antibodies against BVDV−1

**Figure 5:**
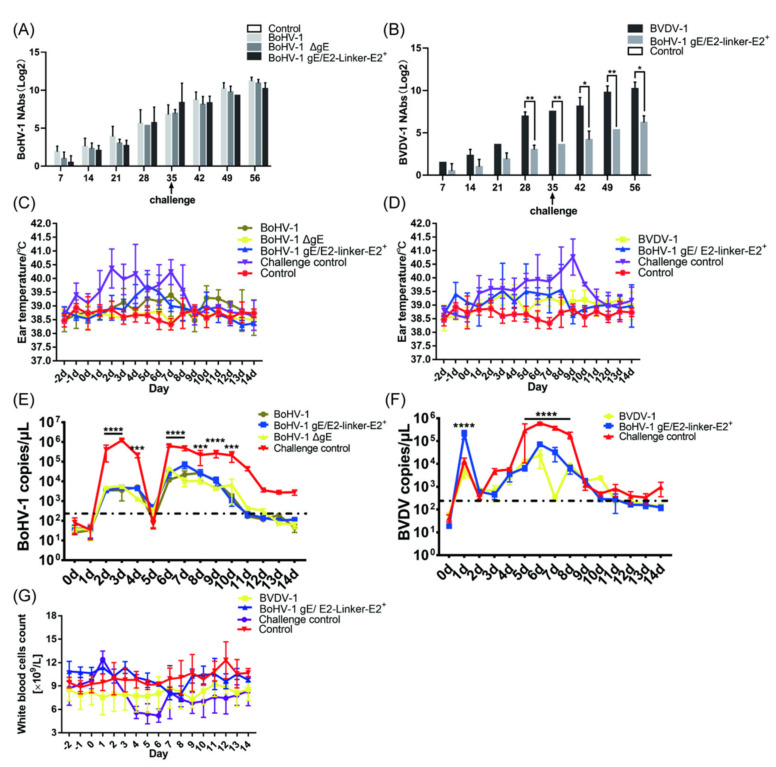
Immunogenicity and serological experiments on BoHV−1 gE/E2−Linker−E2^+^ and BoHV−1 ΔgE on calves. Immunogenicity experiments: inactivated antigen was intramuscularly immunized into the neck, and the second immunization occurred 21 days after the first. Serum samples were collected to detect specific neutralizing antibodies at an interval of 7 days after the initial immunization until day 56. (**A**) BoHV−1 specific neutralizing antibody. (**B**) BVDV−1 specific neutralizing antibody, protective experiment: after 42 d immunization, calves were challenged with BoHV−1 or BVDV−1, respectively. (**C**) Changes in calf body temperature in each group after the BoHV−1 challenge. (**D**) Changes in calf body temperature in each group after the BVDV−1 challenge. (**E**) Viral load in calf nasal swabs after BoHV−1 challenge. (**F**) Viral load in calf anal swabs after BVDV−1 challenge. (**G**) Percentage decrease in the average number of white blood cells in each group of animals after the BVDV−1 challenge. The above experimental data are expressed in mean ± SD. Univariate (ANOVA) was used to analyze whether the difference was significant. *p* < 0.05 is considered significant. Mark * is *p* < 0.05, mark ** is *p* < 0.01, mark *** is *p* < 0.001, mark **** is *p* < 0.0001.

To determine whether there was a difference in immunogenicity between the recombinant virus and its parent virus on calves and whether antibodies produced by BoHV−1 gE/E2−Linker−E2^+^ can neutralize BVDV−1, we mixed inactivated antigens with 206 adjuvants in immune calves. BVDV−1 and BoHV−1 inactivated antigens were used as a positive control, and 206 adjuvants were used as a negative control. On day 7 of immunization, the serum of all calves in the BoHV−1 immunization group became positive, with the highest neutralizing titer reaching 1:4 (Figure 5A). In BoHV−1 gE/E2−Linker−E2^+^ and BoHV−1 ΔgE immunization groups, only two calves’ serum-neutralizing titer reached 1:2. As expected, adjuvants alone did not produce any neutralizing antibodies (Figure 5A). After 21 days, the average serum neutralizing titer of calves in the BoHV−1 immunization group reached 19.96. The average serum-neutralizing titer of the BoHV−1 ΔgE and BoHV−1 gE/E2−Linker−E2^+^ immunization group did not significantly differ from that of the BoHV−1 immunization group. On day 28, the average serum-neutralizing titer of the calf in the BoHV−1 immunization group reached 72.30. On day 42, the average serum-neutralizing titer of the calf in the BoHV−1, BoHV−1 ΔgE, and BoHV−1 gE/E2−Linker−E2^+^ immunization groups reached 489.50, 337.59, and 387.63, respectively (Figure 5A). The final neutralizing titer of the BoHV−1 immunization group was not significantly different from that of the BoHV−1 gE/E2−Linker−E2^+^ and BoHV−1 ΔgE immunization groups. Similarly, BVDV−1 and BoHV−1 gE/E2−Linker−E2^+^ immunization groups became positive in serum 7 days after immunization. The average serum-neutralizing titer of calves after 42 days was highest at 337.59 and 21.82, respectively. The neutralizing titer of the BoHV−1 gE/E2−Linker−E2^+^ immunization group was significantly different from that of the BVDV−1 immunization group (Figure 5B). Importantly, all immunization groups showed intense neutralizing activity against BoHV−1 infection in calves, producing a final titer comparable to the neutralizing titer induced by parent BoHV−1. At the same time, the E2-Linker-E2 protein expressed by BoHV−1 gE/E2−Linker−E2^+^ could cause calves to produce neutralizing antibodies against BVDV−1 infection.

#### 3.4.2. After BoHV−1 and BVDV−1 Challenge, the Time and Level of Viral Shedding of Immunized Calves Were Decreased Compared to Those of the Challenge Control Group

At 3 weeks after the second immunization, BoHV−1, BoHV−1 ΔgE, BoHV−1 gE/E2−Linker−E2^+^, and the challenge control group were challenged with BoHV−1 in the nasal cavity. Three calves were in the challenge control group, two of which (#106, #104) had an increase in body temperature on day 2, and the body temperature of another (#105) started to increase on day 4, reaching 40.7 °C in day 7 (Figure 5C) and showed obvious clinical symptoms such as dyspnea and purulent nasal fluid on days 6 and 7, respectively (Supplementary Tables S1 and S5). On day 4, serous nasal fluid was observed in the nasal cavity for 10 days. After the challenge of BoHV−1, only one calf in the BoHV−1 immunization group experienced an increase in body temperature of 0.1~0.2 °C on days 6, 8, 10, and 13. 2 calves in the BoHV−1 gE/E2−Linker−E2^+^ immunization group experienced an increase in body temperature, one of which rose to 40.2 °C on day 5 and returned to normal the next day. Another calf had a body temperature of 39.7 °C, 39.6 °C, and 40.2 °C on days 4~6, respectively, and returned to normal on day 7. All calf temperatures in the BoHV−1 ΔgE immunization group were within the normal range (38.5 °C~39.5 °C). Nasal swabs were taken from calves in the above immunization groups for antigen detection using BoHV−1 gE−F/R primers and the challenge control group was positive for PCR on day 2. However, until day 12, BoHV−1 was not detected (Supplementary Table S6). We also used fluorescence quantitative primer gB−F/R to detect the viral shedding of BoHV−1 after the challenge. The results showed that the viral shedding level of the immunization group was smaller than that of the challenge control group from day 2~4, the viral shedding level of the challenge control group sharply decreased after day 4, and the viral shedding level sharply increased after day 5, and the viral shedding level of the challenge control group was significantly different from that of the immunization group in day 5~15. The difference in viral shedding on days 3 and 10 was significant, and the immunization group was significantly different from the challenge control group. Excepting day 0, day 1, and day 5, the immunization group was significantly different from the challenge control group in viral shedding (Figure 5E). When all immunization groups were compared with the challenge control group, differences on day 2~4 and day 6~10 were significant.

At 3 weeks after the second immunization, BoHV−1 gE/E2−Linker−E2^+^, BVDV−1, and the control immunization group were challenged with BVDV−1 in the nasal cavity. In the challenge control group, one calf (#124) had an elevated body temperature on day 1, and the other two calves had an elevated temperature on day 4, had watery diarrhea on day 5, and on day 10, the symptoms were reduced, lasting for about 5 days (Figure 5D) (Supplementary Tables S1 and S5). After BVDV−1 challenge, the body temperature of the BVDV−1 immunization group was within the normal range. One calf in the BoHV−1 gE/E2−Linker−E2^+^ immunization group experienced an elevated body temperature, which rose to 40.6 °C on day 3 and returned to the normal range the next day. Body temperature on days 5, 6, 7, and 8 was 40.6 °C, 39.6 °C, 40.0 °C, and 40.6 °C, respectively, and on day 17 it had recovered to within the normal range. The body temperature of all other calves was within the normal range (38.5 °C~39.5 °C). Anal swabs were taken for calves in the above immunization groups for antigen detection using 5′ UTR−F/R primers, and the maximum viral shedding time of one of the calves in the challenge control group was found to be 12 days. The maximum viral shedding time in the BVDV−1 and BoHV−1 gE/E2−Linker−E2^+^ immunization groups was approximately 6 days (Supplement Table S7). We used the fluorescence quantitative primer BVDV−1−F/R to detect viral shedding after the BVDV−1 challenge, and the viral shedding could be detected in the challenge control group from days 1~14. Compared with the BVDV−1 challenge control group, differences in the level of viral shedding in BoHV−1 gE/E2−Linker−E2^+^ and BVDV−1 immunization groups was extremely significant on day 2 and days 5~8. The virus copy number of calves immunized with BoHV−1 gE/E2−Linker−E2^+^ and BVDV−1 inactivated antigens was significantly different from that of the challenge control group. One calf (#121) in the BoHV−1 gE/E2−Linker−E2^+^ immunization group was found to have BVDV−1 in day 11, while BVDV−1 could not be detected in the remaining calves on day 11, as in the BVDV−1 immunization group (Figure 5F).

#### 3.4.3. After the BVDV−1 Challenge, Leukopenia Was Observed in Calves of the Challenge Control Group, While No Leukopenia Was Observed in the Immunization Groups

The total number of white blood cells was measured and recorded 3 days before the challenge (−2, −1, 0 dpc) and 14 days after the challenge. The results showed that the calves in the challenge control group all had symptoms of leukopenia. One calf showed a decreasing number of white blood cells on days 4, 5, 6, 9, 10, and 11, and another showed a decrease in the number of the white blood cells on days 5, 6, 7, 9, 10, 11, and 12, and the last calf showed a decrease in the number of white blood cells on days 4, 5, 6, 7, and 8. It is worth noting that the maximum percentage decrease in white blood cells in the challenge control group of calves was in the range of 25.52%~54.72%, and the average percentage decrease in the group was 35.38% (Figure 5G). According to the percentage decrease in white blood cell count, none of the three calves in the BVDV−1 immunization group had leukopenia (Supplementary Table S8). One of the calves in the BoHV−1 gE/E2−Linker−E2^+^ immunization group had symptoms of leukopenia, and similar symptoms also occurred on day 7 (30.03%) and day 8 (32.91%), lasting until day 9, at which point the calf returned to normal and there was no downward trend on days 9~12. The remaining calves did not differ from the blank control group, and the number of white blood cells decreased from 7.3 × 10^9^ to 7.0 × 10^9^ on day 7, within the normal range. In summary, inoculating BoHV−1 gE/E2−Linker−E2^+^ can protect calves from BVDV−1 infection.

#### 3.4.4. Assessment of Muscle Damage

**Figure 6:**
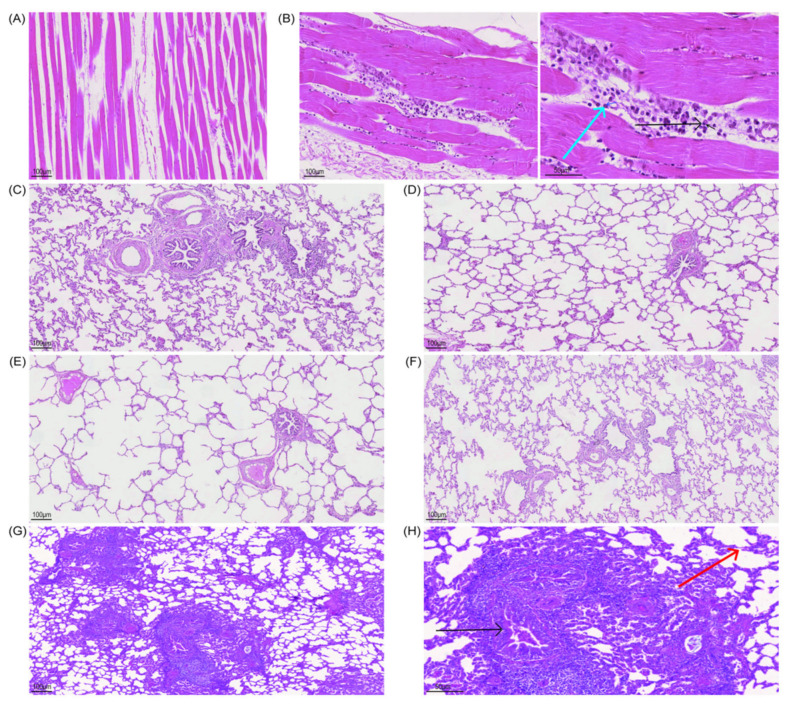
Histopathology of calf lung slices. (**A**) Neck muscles in the blank control group (10 × 10). (**B**) Neck muscles in the immunization group; blue arrows showing macrophages, black arrows showing plasma cells (10 × 10). (**C**) BoHV−1 immunization group (10 × 10). (**D**) BoHV−1 ΔgE immunization group (10 × 10). (**E**) BoHV−1 gE/E2−Linker−E2^+^ immunization group (10 × 10). (**F**) Representative slices of normal lung tissue in the blank control group (10 × 10). (**G**) (10 × 10) and (**H**) (10 × 20) show representative slices of lesion tissue in the challenge control group, and black arrows show bronchi that were compressed and bulging, with bronchospasm and contraction; black arrows show peripheral lymphocytes and monocyte infiltration along with peribronchitis or tubular formations; red arrows show narrowing of alveolar septum and emphysema.

Inactivated antigen immunized in the neck of calves, and muscle tissue was taken from the immune site on day 4 of immunization. The results showed that the blank control group had normal muscle cell structure, striations, and no inflammatory cell infiltration (Figure 6A). In the immunization group, the muscle cells at the neck challenge site were normal in structure, no granulomas had formed, the muscles were striated, macrophages appeared around them, and plasma cell infiltration occurred (Figure 6B). The results showed that the inactivated antigen did not damage the calf local tissues and could lead to a good immune response.

#### 3.4.5. According to the Histopathological Lesions of the Calf Lungs, the Immunization Groups Had a Good Protective Effect, and the Lung Lesions Were Reduced Compared with the Challenge Control Group

After BoHV−1 and BVDV−1 were challenged 14 days later, the calves were euthanized, and pathological sections of the lungs and small intestine were taken. HE staining results showed that, in the challenge control group (Figure 6G), the lung septum of calves was extensively thickened, the alveolar septum widened, the alveoli became smaller, and parenchyma and the bronchi were compressed. There was bulging, bronchospasm, contraction, and varying numbers of surrounding lymphocytes and monocytes infiltrated and proliferated, with peribronchitis or tubular formations, which is a typical symptom of interstitial pneumonia. This was accompanied by a narrowing of the lung septum, emphysema, and severe lesions. BoHV−1 (Figure 6C), BoHV−1 ΔgE (Figure 6D), and BoHV−1 gE/E2−Linker−E2^+^ (Figure 6E) immune groups had mild lesions, clear alveolar spaces, and an intact lung structure, with little difference compared to the blank control group (Figure 6F) (Supplementary Table S10). The lung lesions of calves in the immunization group were significantly different from that of the challenge control group (Supplementary Table S10).

#### 3.4.6. According to the Histopathological Lesions of the Small Intestine of Calves, the Immunization Group Showed a Good Protective Effect, and the Lesions of the Small Intestine Were Reduced Compared to the Challenge Control Group

**Figure 7:**
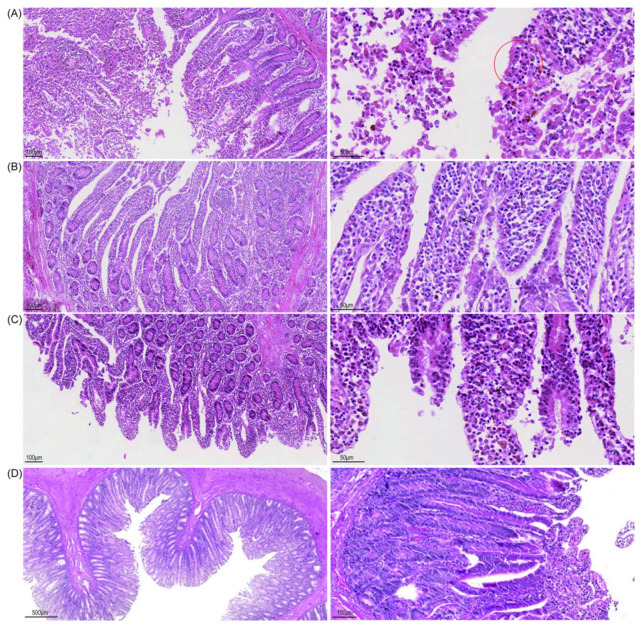
Histopathology of calf small-intestine sections. (**A**) Representative section of lesion tissue in the challenge control group; red circles show apoptosis and necrosis (10 × 10). (**B**) BVDV−1 immunization group (10 × 10). (**C**) BoHV−1 gE/E2−Linker−E2^+^ immunization group (10 × 10). (**D**) Representative section of normal lung tissue in the blank control group (10 × 10).

The results of HE staining of the small intestine showed that in the challenge control group (Figure 7A), the lamina propria of calves was lightly stained and obviously hyperemic, the epithelial cells were exfoliated and necrotic, and the villi structure was incomplete. Lamina propria cells were increased, mainly lymphocytes, macrophages, and acidic granulocytes. BVDV−1 (Figure 7B) and BoHV−1 gE/E2−Linker−E2^+^ (Figure 7C) immunization group lesions were mild, and the overall small intestine structure was intact. Each layer of the intestinal wall was clearly demarcated, and most of the villi structure was intact(Supplementary Table S11). Compared with the blank control group (Figure 7D), there was a large amount of lymphocyte hyperplasia at the bottom of lamina propria and submucosa, which indicates that the immune inactivated antigen can strengthen the immune function of calves. In the BVDV−1 and BoHV−1 gE/E2−Linker−E2^+^ immunization groups, partial epithelial cells shed, allowing for lymphocytes and mononuclear macrophages to exude. The shed cell structure was intact, with suspected physical damage from sampling or changes after calf death. The results showed that the deletion of virulence genes and expression of exogenous proteins did not affect the viral immunogenicity, which could protect calves from BoHV−1 and BVDV−1 (Supplementary Table S10).

## 4. Discussion

BoHV−1 and BVDV have led to significant economic losses for the global cattle industry. Vaccination is an effective measure to prevent infection, and some countries are working to control or eradicate the infection [43]. To date, some countries have used conventional modified live vaccines (MLV), killed-virus vaccine (KV), and subunit vaccines to control BoHV−1, and some European countries have used labeled vaccines to prevent and control BoHV−1 infection [44]. These labeled vaccines usually have fewer antigenic proteins than the parent virus, typically glycoproteins of viral genes or related enzymes for synthesizing, and the deletion of these antigenic proteins can be used to detect antibody responses to specific deletion proteins. This could help to distinguish wild virus infections from vaccinated animals [45]. In marker vaccines lacking the gE gene, which is responsible for synthesizing BoHV−1 glycoprotein E (gE), this glycoprotein and glycoprotein I (gI) form a heterodimer complex to form the Fc receptor and can bind to the Fc domain of IgG, thereby disrupting the host’s immune response and causing immunosuppression. The use of gE deletion labeled vaccines, which can serologically distinguish between vaccinated and infected animals, has been included in BoHV−1 control programs by European countries [46]. BVDV seroprevalence rate is high in China. Therefore, the prevention and control of this disease should be strengthened [14,47]. Based on data analysis from the periods 2003~2009 and 2010~2018, the seroprevalence rate of BVDV in cattle was 57%, and the positive rate of viral RNA was estimated at 27.1% [47]. The direct economic loss per cattle head ranges 0.5 to 687.8 US dollars. In addition, BVDV causes enormous damage to the host’s immune system, and its mediated immunosuppression makes calves more susceptible to other infectious diseases. If the cow is infected with BVDV in early pregnancy, it can give birth to calves with persistent infection (PI) who are infected and detoxified for life, posing challenges to the prevention and control of BVDV [48]. Persistently infected animals are usually more likely to transmit BVDV than acutely infected animals because they can shed the virus over a lifetime, and many countries worldwide are achieving population purification by screening and phasing out PI cattle [49,50].

At present, vaccines against BRDC include trivalent attenuated BoHV−1, BVDV−1, and BVDV−2 live vaccines, BoHV−1 live attenuated vaccines, and BoHV−1/BVDV bivalent live attenuated vaccines. Although these vaccines can prevent BoHV−1 and BVDV infections, due to the incubation period in the trigeminal ganglia of the traditional BoHV−1 (MLV) vaccine, external environmental stress can cause the virus to reactivate and detoxify from the nasal cavity, causing miscarriages in the dairy industry (BoHV−1), respiratory diseases in the beef cattle and dairy industry (BoHV−1 and BVDV), and persistent infections in the dairy industry (BVDV). Therefore, only BoHV−1 labeled vaccines lacking the virulence gene gE are allowed in several EU countries. Under pressure from the external environment, when using a BoHV−1 labeled vaccine that lacks the virulence gene gE, the virus does not spread from the nasal cavity of the challenged animal after it is reactivated during the incubation period. The use of live attenuated BVDV as a vaccine is suspected to cause problems associated with wtBVDV infection in the cattle industry [51,52,53], making young herds vulnerable to severe and widespread BVDV infection. The recombinant baculovirus vaccine for E2 has been commercially produced and combined with inactivated BVDV in a goat model to enhance the immune protection of animals against virulent BVDV challenges, which achieved a good protection effect [54].

Genetically engineered subunit vaccines are generally less immunogenic than traditional live attenuated and inactivated vaccines. To increase the expression of E2 protein in the vector BoHV−1, we constructed two mutant viruses in this study, BoHV−1 gE/E2−Linker−E2^+^ and BoHV−1 ΔgE, both of which deleted the gE virulence gene. In addition, BoHV−1 gE/E2−Linker−E2^+^ at the gE gene location was inserted into our optimized BVDV−1 E2 gene and combined with the BoHV−1 gD signal peptide sequence. The final protein expression of BoHV−1 gE/E2−Linker−E2^+^ can reach 66% of that of BVDV−1. In this study, two E2 proteins were expressed in tandem by codon optimization and the addition of signal peptide sequences, which increased the expression of E2 proteins to a certain extent. In addition, Xu et al. used a prokaryotic expression system to express the E2 protein of the Changchun 184 strain, and the expressed protein accounted for 6.25% of the bacterial protein, but the E2 protein expressed after removing the transmembrane region of the E2 gene accounted for 35.7% of the bacterial protein. The results showed that BoHV−1 could highly express BVDV E2 protein. Similar to the findings of Schmitt et al., pestivirus glycoproteins can be efficiently expressed and incorporated into the viral envelope by BoHV−1, which is a suitable vector for expressing BVDV E2, and the conformation is similar to the E2 protein expressed by BVDV [55]. Notably, the E2 protein expressed by BoHV−1 gE/E2−Linker−E2^+^ allowed guinea pigs to produce serum-neutralizing antibodies against BVDV−1 infection. Similar to previous studies, Bhuyanaa et al. constructed a recombinant Lactobacillus casei pELX1-E2 to express the E2 gene of BVDV and evaluated the immune response elicited in mice, revealing that the recombinant strain could induce high levels of E2-specific antibodies [56]. However, after 28 days, the neutralizing antibody produced by BoHV−1 gE/E2−Linker−E2^+^ immunization group was significantly different from that of the BVDV−1 immunization group. We speculate that the inactivated virus does not have replication properties, which makes the protein amount of BoHV−1 gE/E2−Linker−E2^+^ different from that of BVDV−1. We found that the final neutralizing antibody produced by BoHV−1 gE/E2−Linker−E2^+^ immunization group was significantly different from that of the BVDV−1 immunization group, but still protected guinea pigs from BVDV−1 infections (Supplementary Table S9). If the average neutralizing antibody titer was only 11.75, it could protect the animals from BoHV−1 and BVDV−1 re-infection and significantly reduce the viral shedding.

Next, to determine whether the above-mentioned recombinant virus immune calves can provide immune protection against BoHV−1 and BVDV−1, we immunized calves with BoHV−1 ΔgE, BoHV−1 and BoHV−1 gE/E2−Linker−E2^+^. On day 28, after the second immunization, the calves challenged with BoHV−1, BoHV−1 ΔgE, BoHV−1 gE/E2−Linker−E2^+^, and BoHV−1-specific serum-neutralizing antibodies increased by four times, and there was no significant difference when compared to the parental virus BoHV−1, indicating that the expression of E2 protein and the deletion virulence gene gE did not affect its immunogenicity. After the BoHV−1 challenge, except for one calf in the BoHV−1 gE/E2−Linker−E2^+^ immunization group, whose body temperature rose to 40.2 °C on day 5 (and returned to normal the next day), the body temperature of the other calves in the immunization groups did not increase. In addition, combined with the results of lung pathology sections, we found that lung lesions were significantly reduced in calves immunized with BoHV−1 ΔgE and BoHV−1 gE/E2−Linker−E2^+^ (Supplementary Table S10). BoHV−1 ΔgE and BoHV−1 gE/E2−Linker−E2^+^ not only protected calves from respiratory symptoms caused by BoHV−1 infection but also reduced associated pathological features (Supplementary Table S5), and the differences were not significant compared to the parental virus (Supplementary Tables S5 and S10). Previous studies have shown that immunizing cattle with recombinant plasmids containing the E2 gene produces an immune response but does not provide complete protection after challenge [22]. However, our study shows that compared with BVDV−1, BoHV−1 gE/E2−Linker−E2^+^ can induce specific neutralizing antibody response to BVDV−1 and reduce the risk of BVDV−1 infection in calves. Based on this result, we speculate that the main reason may be that the two optimized E2s expressed in this study increased the protein expression or produced partial homodimers, which is consistent with the recent studies on live attenuated vaccines [40].

In contrast, we codon-optimized the BVDV−1 E2 gene and added a signal peptide sequence of the BoHV−1 gD gene to express two E2 proteins in tandem at the gE gene site, which could protect calves from the risk of BVDV−1 infection. Pecora realized the fusion expression of E2 protein and single-chain antibody against BVDV−1 and BVDV−2 using an insect cell expression system. The results showed that both calves and guinea pigs could produce better levels of E2 antibodies after immunization, and both had good protection after being challenged with two types of viruses. The results of this study are similar to those mentioned above [57]. We believe that BoHV−1 gE/E2−Linker−E2^+^ can both protect calves from BoHV−1 infection and BVDV−1 infection. Furthermore, this study demonstrates the safety of all the above recombinant viruses in guinea pig and calf models. The protein expressing the viral vector has high immunogenicity, which demonstrates the potential of BoHV−1 gE/E2−Linker−E2^+^ and BoHV−1 ΔgE as vaccine candidates. In conclusion, by using BoHV−1 gE/E2−Linker−E2^+^ inactivated antigens, we achieved the same level of protection against two viruses at the same time and avoided the side effects associated with BoHV−1 and BVDV vaccines. In addition, from the production perspective, the vaccine is low-cost, and only one virus needs to be cultivated for the vaccine, which is more economical. Moreover, the vaccine has higher safety compared to the live attenuated vaccine. These attenuated viruses have established a life-long incubation period that may be reactivated by external environmental stimuli, and the diseased cattle may become the source of infection, causing the virus to spread in the herd. Moreover, the immunized BoHV−1 gE/E2−Linker−E2^+^ inactivated antigens can distinguish vaccinated from wild-virus-infected calves, laying the foundation for future clarity.

## 5. Conclusions

At present, live polyvalent vaccines that are modified against BoHV−1 and BVDV have limitations in terms of their safety and efficacy. In this study, the gene-engineered vector subunit vaccine (BoHV−1 gE/E2−Linker−E2^+^) and the BoHV−1 ΔgE gene deletion vaccine had the advantages of safety and a remarkable effect on the prevention of BVDV−1 and BoHV−1. In our study, the results demonstrate that tandem expression of the two optimized E2 proteins, in combination with the gD gene signal peptide, can increase the expression of the BVDV−1 E2 protein, protecting calves from BVDV−1 infection. Therefore, we believe that the BoHV−1-gE/E2-linker-E2^+^ genetically engineered subunit vaccine will be a substitute to the current BoHV−1 and BVDV−1 prophylaxis as a conventional bivalent vaccine.

## Figures and Tables

**Table 1:** Primers, sgRNAs and restriction sites were used in this study.

Primers	Nucleotide Sequences (5′–3′)	Genome Position	Restriction Sites
LgE−F	aagcttTGCTCTTCTCCATCGCCCATC	120663–120683	*Hind* III
LgE−R	ggtaccCATTGCCAAATGCCCTTTTCGA	121695–121716	*Kpn* I
EGFP−F	ggtaccATGGTGAGCAAGGGCGA	1–17	*Kpn* I
EGFP−R	ggatccCTTGTACAGCTCGTCC	702–717	*BamH* I
RgE−F	ggatccAGTCGTTACTTCGGACCGTTTGGTGC	122847–122872	*BamH* I
RgE−R	gaattcTCAGCGCCTCGATAGTTTTCGTTGAC	123569–123594	*EcoR* I
R1gE−F	ggatccCTCAAGTCCATCCTCCGCTAG	123421–123441	*BamH* I
R1gE−R	gaattcGCCCTTGTCATATTTTTTTAA	124486–124506	*EcoR* I
BoHV−1 gE−F	CGCCGGGTTGTTAAATGGGTCTCG	121573–121596	
BoHV−1 gE−R	CGGGCGCGTCCTCGATGGTG	123664–123683	
pX459−EGFP−sgRNA1−F	caccgGTCGCCCTCGAACTTCACCT	335–354	*Bbs* I
pX459−EGFP−sgRNA1−R	aaacAGGTGAAGTTCGAGGGCGACc	335–354	*Bbs* I
pX459−EGFP−sgRNA2−F	caccgGTGGTTGTCGGGCAGCAGCA	581–600	*Bbs* I
pX459−EGFP−sgRNA2−R	aaacTGCTGCTGCCCGACAACCACCc	581–600	*Bbs* I
pX459−gE−sgRNA1−F	caccgCGGCGACGAGGAGACGCAGTTGG	122217–122239	*Bbs* I
pX459−gE−sgRNA1−R	aaacCCAACTGCGTCTCCTCGTCGCCGc	122217–122239	*Bbs* I
pX459−gE−sgRNA2−F	caccgCGCCGATGAGCCGGTCGTACAGG	122190–122212	*Bbs* I
pX459−gE−sgRNA2−R	aaacCCTGTACGACCGGCTCATCGGCGc	122190–122212	*Bbs* I
E2−F	cggggtaccATGCAAGGGCCGACATTGGCCG	1–22	*Kpn* I
E2−Rm	GCCGCTGCCGCCGCTGCCCTACCCGGCCACGACCA	1194–1128	
E2−Fm	GGCAGCGGCGGCAGCGGCATGCAAGGGCCGACATT	1111–1145	
E2−R	cgcggatccCTACCCGGCCACGACCACCAC	2218–2238	*BamH* I
BVDV−F	GGGNAGTCGTCARTGGTTCG	177–196	
BVDV−R	GTGCCATGTACAGCAGAGWTTTT	354–376	
Probe	FAM-CCAYGTGGACGAGGGCAYGC-TAMRA	224–243	
gB−F	CGTGACGGTAGCCTGGGACT	56473–56492	
gB−R	CGTCTCGCAGCATTTCGTC	56543–56561	
E2−E2−F	GCGTTTCAGATGGTGTGC	373–390	
E2−E2−R	CTTGCTGCGGCGGTAGGT	463–480	
5′UTR−F	TAGCCATGCCCTTAGTAGGAC	93–113	
5′UTR−R	CTCCATGTGCCATGTACAGCA	362–382	

LgE-F/R amplified left homologous arm. RgE-F/R and R1gE-F/R amplified two right homologous arms. BoHV−1 gE−F/R identification mutation viruses. pX459-EGFP-sgRNA1−F/R and pX459-EGFP-sgRNA2−F/R were sgRNAs designed for EGFP. pX459−gE−sgRNA1−F/R and pX459−gE−sgRNA2−F/R were sgRNAs designed for the BoHV−1 gE gene. EGFP-F/R was used to amplify the EGFP gene. E2F/Rm, E2Fm/R was used to amplify the E2-linker-E2 gene. BVDV−F/R was a fluorescence quantitative primer sequence for BVDV−1. gB−F/R was a fluorescence quantitative primer sequence for the BoHV−1 gB gene. E2-E2−F/R was a fluorescence quantitative primer sequence of the E2 gene of recombinant BoHV−1 gE/E2−Linker−E2^+^. The 5′UTR−F/R was a BVDV−1 detection primer.

## Data Availability

The data presented in this study are available on request from the corresponding author.

## Supplementary Materials

The following supporting information can be downloaded at: https://www.mdpi.com/article/10.3390/v14081618/s1, Table S1: Clinical Scoring Criteria. Table S2: BoHV−1 shedding of nasal swabs in guinea pigs after challenge. Table S3: BVDV−1 shedding of anal swabs in guinea pigs after challenge. Table S4: White blood cell count in guinea pigs after challenge. Table S5: Clinical score in calves after challenge. Table S6: BoHV−1 shedding of nasal swabs in calves after challenge. Table S7: BVDV−1 shedding of nasal swabs in calves after challenge. Table S8: White blood cell count in calves after challenge. Table S9: Lung histology score in guinea pigs after challenge BoHV−1. Table S10: Lung histology score in calves after challenge BoHV−1. Table S11: Small intestine histology score in calves after challenge BVDV−1.
