# Supplementary material for: Recombinant Bovine Herpesvirus Type I Expressing the Bovine Viral Diarrhea Virus E2 Protein Could Effectively Prevent Infection by Two Viruses
Source: Viruses. 2022 Jul 25;14(8):1618. doi: 10.3390/v14081618 (PMC9331970; doi:10.3390/v14081618)
Supplement: Supplementary file 1 [file viruses-14-01618-s001.zip › viruses-1767460-supplementary.pdf]

Supplemental Table S1. Clinical Scoring Criteria

Clinical score	Rectal temperature	Respiratory symptoms	Mental state	Nasal secretions	Diarrhea symptoms
Normal	≤39.5℃	Normal breathing	Normal status	none	Normal stool
Mild	39.6~40.0℃	slight difficulty breathing	Head down, moving infrequently	Serous nasal fluid	Stools are soft and slightly runny
Moderate	40.1~40.5℃	Dyspnea, obvious abdominal breathing, with cough	Lying down, reluctance to move purulent nasal fluid	Purulent nasal fluid	Watery diarrhea
Severe	40.6~41.5℃	Grunting or harsh breathing	Difficulty standing and unresponsive to stimuli from people and the environment	Massive mucopurulent nasal fluid	Watery bloody diarrhea

Supplemental Table S2. BoHV-1 shedding of nasal swabs in guinea pigs after challenge

Group and guinea pigs number \ Challenge days		0	1	2	3	4	5	6	7	8	9	10	11	12	13	14
Challenge control group	25	-	-	-	-	-	-	-	-	-	-	-	-	-	-	-
	26	-	-	-	-	-	-	-	-	-	-	-	-	-	-	-
	27	-	-	-	-	-	-	-	-	-	-	-	-	-	-	-
	28	-	-	-	-	+	-	-	-	-	-	-	-	-	-	-
	29	-	-	-	-	-	-	-	-	-	-	-	-	-	-	-
	30	-	-	-	-	-	-	-	-	-	-	-	-	-	-	-
BoHV-1 immunization group	31	-	-	-	-	-	-	-	-	-	-	-	-	-	-	-
	32	-	-	-	-	-	-	-	-	-	-	-	-	-	-	-
	33	-	-	-	-	-	-	-	-	-	-	-	-	-	-	-
	34	-	-	-	-	-	-	-	-	-	-	-	-	-	-	-
	35	-	-	-	-	-	-	-	-	-	-	-	-	-	-	-
	36	-	-	-	-	-	-	-	-	-	-	-	-	-	-	-
BoHV-1 ΔgE immunization group	37	-	-	-	-	-	-	-	-	-	-	-	-	-	-	-
	38	-	-	-	-	-	-	-	-	-	-	-	-	-	-	-
	39	-	-	-	-	-	-	-	-	-	-	-	-	-	-	-
	40	-	-	-	-	-	-	-	-	-	-	-	-	-	-	-
	41	-	-	-	-	-	-	-	-	-	-	-	-	-	-	-
	42	-	-	-	-	-	-	-	-	-	-	-	-	-	-	-
BoHV-1 gE/E2-Linker-E2^{+} immunization group	43	-	-	-	-	-	-	-	-	-	-	-	-	-	-	-
	44	-	-	-	-	-	-	-	-	-	-	-	-	-	-	-
	45	-	-	-	-	-	-	-	-	-	-	-	-	-	-	-
	46	-	-	-	-	-	-	-	-	-	-	-	-	-	-	-
	47	-	-	-	-	-	-	-	-	-	-	-	-	-	-	-
	48	-	-	-	-	-	-	-	-	-	-	-	-	-	-	-

Blank control group	19	-	-	-	-	-	-	-	-	-	-	-	-	-	-	-
	20	-	-	-	-	-	-	-	-	-	-	-	-	-	-	-
	21	-	-	-	-	-	-	-	-	-	-	-	-	-	-	-

Supplemental Table S3. BVDV-1 shedding of anal swabs in guinea pigs after challenge

Group and guinea pigs number		Challenge days 0	1	2	3	4	5	6	7	8	9	10	11	12	13	14
BVDV-1 immunization group	1	-	+	+	+	+	+	+	-	-	-	-	-	-	-	-
	2	-	+	+	-	+	+	-	-	-	-	-	-	-	-	-
	3	-	+	+	+	+	+	+	-	-	-	-	-	-	-	-
	4	-	+	+	+	+	-	-	-	-	-	-	-	-	-	-
	5	-	+	+	+	+	+	+	-	-	-	-	-	-	-	-
	6	-	+	+	-	+	+	+	-	-	-	-	-	-	-	-
BoHV-1 gE/E2-Linker-E2^{+} immunization group	7	-	+	+	-	+	+	+	+	-	-	-	-	-	-	-
	8	-	+	+	+	+	+	+	+	-	-	-	-	-	-	-
	9	-	+	+	+	-	+	-	+	-	+	-	-	-	-	-
	10	-	+	+	+	+	+	+	+	+	-	-	-	-	-	-
	11	-	+	+	+	+	+	+	+	+	+	+	-	-	-	-
	12	-	+	+	+	+	+	+	+	+	+	+	-	-	-	-
Challenge control group	13	+	+	+	+	+	+	+	+	+	+	+	+	+	+	+
	14	+	+	+	+	+	+	+	+	+	+	+	+	+	+	+
	15	+	+	+	+	+	+	+	+	+	+	+	+	+	+	+
	16	+	+	+	+	+	+	+	+	+	+	+	+	+	+	+
	17	+	+	+	+	+	+	+	+	+	+	+	+	+	+	+
	18	+	+	+	+	+	+	+	+	+	+	+	+	+	+	+
Blank control group	19	-	-	-	-	-	-	-	-	-	-	-	-	-	-	-
	20	-	-	-	-	-	-	-	-	-	-	-	-	-	-	-
	21	-	-	-	-	-	-	-	-	-	-	-	-	-	-	-

Supplemental Table S4. White blood cell count in guinea pigs after challenge

Group and guinea pigs number \ Challenge days		0	1	2	3	4	5	6	7	8	9	10	11	12	13	14	15
BVDV-1 immunization group	1	7.4	6.1	7.3	7.6	6.3	6.8	6.8	6.8	7.0	7.4	6.5	7.1	7.9	8.8	8.8	8.3
	2	5.2	7.8	6.6	5.8	7.2	4.9	5.8	7.2	8.6	6.5	6.0	7.2	9.0	6.5	8.8	10.0
	3	8.0	7.7	7.3	7.0	7.1	6.7	6.8	6.9	6.7	7.1	7.5	7.3	6.9	7.9	8.0	6.6
	4	7.6	6.5	7.2	8.0	7.2	6.9	6.7	7.2	7.6	8.2	6.3	6.4	7.0	6.7	6.2	7.3
	5	6.8	7.2	7.0	7.3	6.8	6.0	6.4	6.8	7.1	6.7	7.5	6.9	6.3	6.4	6.2	6.0
	6	8.3	7.9	6.9	6.9	7.2	8.2	7.6	6.9	7.9	7.4	7.6	8.6	8.4	8.2	7.2	7.5
BoHV-1 gE/E2-Linker-E2$^+$ immunization group	7	7.6	8.0	8.5	7.7	8.3	7.0	7.6	8.6	8.4	7.9	7.6	10.9	8.1	8.5	7.8	8.4
	8	8.5	8.6	8.6	7.6	7.3	6.4	7.2	7.1	8.6	8.1	7.8	7.8	7.6	8.2	7.9	8.6
	9	9.1	8.2	10.6	10.1	9.2	9.3	8.5	9.0	9.0	7.7	8.6	8.2	9.6	8.8	8.3	8.7
	10	7.6	7.2	6.9	6.9	7.1	7.2	8.1	7.7	7.4	7.3	6.9	8.2	7.7	7.9	7.5	7.4
	11	8.2	7.4	7.1	8.2	8.0	7.5	7.6	7.8	7.4	6.9	6.8	7.0	7.0	7.2	7.5	7.7
	12	8.1	7.6	6.9	8.0	6.9	6.8	7.2	7.5	7.9	6.9	7.4	7.0	6.8	6.7	7.1	8.0
Challenge control group	13	7.5	6.9	8.2	8.5	8.3	8.2	6.0	6.2	6.4	5.8	8.5	9.7	6.6	6.7	7.1	7.6
	14	6.3	7.2	7.5	7.6	10.3	7.7	5.8	5.7	5.5	5.5	8.2	6.8	7.5	6.4	6.9	8.0
	15	8.2	6.5	7.2	6.3	9.7	8.0	6.1	4.5	7.2	6.5	8.3	7.8	9.4	7.9	8.1	6.6
	16	7.7	7.4	7.0	7.7	7.5	8.1	6.2	6.2	6.2	6.0	6.9	7.1	7.7	7.4	7.3	7.8
	17	7.6	8.0	6.8	7.2	7.4	6.9	6.2	6.4	6.4	6.5	6.8	6.8	7.2	7.4	7.4	7.4
	18	6.9	7.5	7.3	8.0	8.1	7.6	6.0	6.1	6.1	7.3	6.5	6.9	7.0	7.0	7.2	7.2
Blank control group	19	8.1	8.5	8.1	9.6	8.3	7.4	7.2	8.2	7.3	7.2	9.3	7.5	10.1	10.1	10.5	10.5
	20	5.6	5.1	6.4	6.2	6.5	5.3	6.1	7.5	6.6	7.2	5.9	5.8	6.2	8.3	8.5	6.6
	21	9.7	8.7	9.0	9.2	9.1	7.8	9.0	6.6	6.9	6.1	6.7	5.4	9.9	9.1	10.0	9.2

Supplemental Table S5. clinical score in calves after challenge

Group and calves number		Challenge days	-2	-1	0	1	2	3	4	5	6	7	8	9	10	11	12	13	14
Control	Blank control group	101	0	0	0	0	0	0	1	0	0	0	0	0	0	0	0	0	0
		102	0	0	0	0	0	0	0	0	0	1	0	0	0	0	0	0	0
		103	0	0	0	0	0	0	0	0	0	0	0	0	0	0	0	0	0
BoHV-1	Challenge control group	104	0	0	0	0	3	2	2	2	2	3	2	2	2	2	2	2	2
		105	0	0	0	0	0	0	2	2	2	2	4	3	3	3	3	2	0
		106	0	0	0	0	2	0	0	0	3	3	3	4	4	3	3	3	2
	BoHV-1 immunization group	107	0	0	0	0	0	0	0	0	0	0	0	0	0	0	0	0	0
		108	0	0	0	0	0	0	0	0	1	0	1	0	1	0	0	1	0
		109	0	0	0	0	0	0	0	0	0	0	0	0	0	0	0	0	0
	BoHV-1 ΔgE immunization group	110	0	0	0	0	0	0	0	0	0	0	0	0	0	0	0	0	0
		111	0	0	0	0	0	0	0	0	1	0	0	0	0	0	0	0	0
		112	0	0	0	0	0	0	0	0	0	0	0	0	0	0	0	0	0
	BoHV-1 gE/E2-Linker-E2+ immunization group	113	0	0	0	0	0	0	0	2	0	0	0	0	0	0	0	0	0
		114	0	0	0	0	0	0	1	1	2	0	0	0	0	0	0	0	0
		115	0	0	0	0	0	0	0	0	0	0	0	0	0	0	0	0	0
BVDV-1	BVDV-1 immunization group	116	0	0	0	0	0	0	0	0	0	0	0	0	0	0	0	0	0
		117	0	0	0	0	0	0	0	0	0	0	1	0	1	0	0	0	0
		118	0	0	0	0	0	0	0	0	0	0	0	1	1	0	0	0	0
	BoHV-1 gE/E2-Linker-E2+ immunization group	119	0	0	0	0	0	0	0	0	0	0	0	0	0	0	0	0	0
		120	0	0	0	0	0	2	0	1	0	1	0	0	1	0	0	0	0
		121	0	0	0	0	0	0	0	0	0	0	0	2	2	1	0	0	0
	Challenge control group	122	0	0	0	0	0	0	2	2	3	3	3	3	2	3	2	2	2
		123	0	0	0	0	0	0	2	3	3	3	4	4	2	2	3	2	2
		124	0	0	0	0	0	0	2	2	3	3	3	2	3	2	2	0	0

Supplemental Table S6. BoHV-1 shedding of nasal swabs in calves after challenge

Group and calves number (Challenge days)		0	1	2	3	4	5	6	7	8	9	10	11	12	13	14
Challenge control group	104	-	-	+	+	+	-	+	-	+	+	+	+	-	-	-
	105	-	-	+	+	+	-	+	+	+	+	+	+	-	-	-
	106	-	-	+	+	+	-	+	+	+	-	+	+	-	-	-
BoHV-1 immunization group	107	-	-	-	-	-	+	+	+	+	+	-	-	-	-	-
	108	-	-	-	-	-	-	-	+	+	+	-	-	-	-	-
	109	-	-	-	-	-	-	+	+	+	+	-	-	-	-	-
BoHV-1 ΔgE immunization group	110	-	-	-	-	-	-	+	+	+	+	-	-	-	-	-
	111	-	-	-	-	-	-	+	+	+	-	-	-	-	-	-
	112	-	-	-	-	-	-	+	+	+	+	-	-	-	-	-
BoHV-1 gE/E2-Linker-$E2^{+}$ immunization group	113	-	-	-	-	-	-	+	+	+	-	+	-	-	-	-
	114	-	-	-	-	-	-	+	+	+	+	-	-	-	-	-
	115	-	-	-	-	-	-	+	+	-	-	-	-	-	-	-

Supplemental Table S7. BVDV-1 shedding of nasal swabs in calves after challenge

Group and calves number	Challenge days	0	1	2	3	4	5	6	7	8	9	10	11	12	13	14
BVDV-1 immunization group	116	-	+	-	-	-	-	-	-	+	+	-	+	-	-	-
	117	-	-	-	-	-	-	-	-	-	+	+	+	-	-	-
	118	-	+	-	-	-	-	-	-	-	+	-	-	+	-	-
BoHV-1 gE/E2-Linker-E2^{+} immunization group	119	-	+	-	-	-	+	+	+	+	+	-	-	-	-	-
	120	-	-	-	-	+	+	+	+	+	+	-	-	-	-	-
	121	-	+	-	-	-	+	+	+	+	+	-	-	-	-	-
Challenge control group	122	-	-	-	-	+	+	+	+	+	+	-	-	-	-	-
	123	-	-	-	-	+	+	+	+	+	+	-	+	-	+	+
	124	-	+	-	+	-	+	+	+	+	-	+	+	-	-	-

Supplemental Table S8. White blood cell count in calves after challenge

Group and calves number \ Challenge days		0	1	2	3	4	5	6	7	8	9	10	11	12	13	14	15	16
BVDV-1 immunization group	116	10.1	10.1	10.5	11.5	10.2	8.2	4.7	4.9	5.1	12.0	8.3	7.4	7.2	7.5	8.5	8.1	9.6
	117	6.2	8.3	8.5	13.6	10.3	7.7	6.0	4.5	4.4	5.4	6.5	5.3	4.9	5.6	5.1	6.4	6.2
	118	9.9	9.1	10.0	12.1	9.7	8.0	6.1	6.9	6.1	7.2	7.0	7.8	9.0	9.7	8.7	9.0	9.2
BoHV-1 gE/E2-Linker-E2+ immunization group	119	7.4	6.1	7.3	5.6	6.3	6.8	6.8	6.8	7.0	7.4	6.5	7.1	7.9	8.8	8.8	8.3	9.2
	120	10.2	9.8	10.2	10.0	10.5	10.2	10.1	10.1	10.5	11.8	10.6	8.5	10.2	11.0	9.6	9.7	9.6
	121	8.0	7.7	7.3	7.0	7.1	6.7	6.3	6.2	6.7	7.1	7.5	6.3	6.9	7.9	8.0	6.3	6.9
Challenge control group	122	10.5	10.6	10.2	11.5	9.7	12.1	10.5	9.2	9.2	7.3	7.0	9.7	10.5	8.6	10.6	9.8	9.7
	123	12.3	10.2	11.5	10.5	9.8	11.4	9.8	10.8	9.2	8.9	9.0	9.8	11.5	10.4	9.5	11.2	9.6
	124	9.8	11.5	10.2	12.1	11.3	10.6	10.0	9.2	9.3	7.9	8.0	11.3	9.2	12.6	8.7	10.6	10.0
Blank control group	101	10.2	8.3	8.6	9.5	8.5	8.6	10.6	8.7	9.2	9.1	8.8	9.3	9.9	10.6	12.6	12.5	10.2
	102	8.9	8.4	8.6	8.4	9.0	9.2	8.6	8.9	9.2	9.1	8.8	9.3	9.1	12.3	14.5	10.3	11.3
	103	9.2	9.8	10.5	10.5	12.3	11.5	10.2	9.8	9.3	11.5	12.6	13.4	10.5	9.8	9.8	8.9	10.5

Supplemental Table S9. Lung Histology score in guinea pigs after challenge BoHV-1

Group and guinea pig number (Pathological changes)		Bronchi: Percentage of infiltrated sites	Bronchi: Degree of infiltration	Interstitial pneumonia
Challenge control group	25	2	2	3
	26	3	3	3
	27	2	1	2
	28	2	2	2
	29	2	2	2
	30	1	1	1
BoHV-1 immunization group	31	1	0	1
	32	1	0	0
	33	0	0	0
	34	1	1	0
	35	0	0	0
	36	0	0	0
BoHV-1 ΔgE immunization group	37	0	0	1
	38	0	0	1
	39	0	0	0
	40	0	0	0
	41	1	2	1
	42	0	1	1
BoHV-1 gE/E2-Linker-E2^{+} immunization group	43	0	0	0
	44	0	0	0
	45	0	0	0
	46	1	0	1
	47	1	0	1
	48	1	0	0
Blank control group	19	1	0	0
	20	0	0	0

	21	1	0	0

Supplemental Table S10. Lung Histology score in calves after challenge BoHV-1

Group and calf number (Pathological changes)		Bronchi: Percentage of infiltrated sites	Bronchi: Degree of infiltration	Interstitial pneumonia
Challenge control group	104	3	2	2
	105	2	2	2
	106	1	2	1
BoHV-1 immunization group	107	0	0	0
	108	1	1	0
	109	0	0	0
BoHV-1 ΔgE immunization group	110	0	1	0
	111	0	0	0
	112	0	0	0
BoHV-1 gE/E2-Linker-E2^{+} immunization group	113	0	0	0
	114	0	0	0
	115	0	0	0

Supplemental Table S11. Small intestine Histology score in calves after challenge BVDV-1

Group and calf number \ Pathological changes		Small intestine
Challenge control group	122	2
	123	3
	124	3
BVDV-1 immunization group	116	0
	117	0
	118	1
BoHV-1 gE/E2-Linker-E2^{+} immunization group	119	0
	120	1
	121	1
